# ER Stress-Induced Clearance of Misfolded GPI-Anchored Proteins via the Secretory Pathway

**DOI:** 10.1016/j.cell.2014.06.026

**Published:** 2014-07-31

**Authors:** Prasanna Satpute-Krishnan, Monica Ajinkya, Savithri Bhat, Eisuke Itakura, Ramanujan S. Hegde, Jennifer Lippincott-Schwartz

**Affiliations:** 1 Cell Biology and Metabolism Program, Eunice Kennedy Shriver National Institute of Child Health and Human Development, Bethesda, MD 20892, USA; 2 Medical Research Council Laboratory of Molecular Biology, Francis Crick Avenue, Cambridge CB2 0QH, UK

## Abstract

Proteins destined for the cell surface are first assessed in the endoplasmic reticulum (ER) for proper folding before release into the secretory pathway. This ensures that defective proteins are normally prevented from entering the extracellular environment, where they could be disruptive. Here, we report that, when ER folding capacity is saturated during stress, misfolded glycosylphosphatidylinositol-anchored proteins dissociate from resident ER chaperones, engage export receptors, and quantitatively leave the ER via vesicular transport to the Golgi. Clearance from the ER commences within minutes of acute ER stress, before the transcriptional component of the unfolded protein response is activated. These aberrant proteins then access the cell surface transiently before destruction in lysosomes. Inhibiting this stress-induced pathway by depleting the ER-export receptors leads to aggregation of the ER-retained misfolded protein. Thus, this rapid response alleviates the elevated burden of misfolded proteins in the ER at the onset of ER stress, promoting protein homeostasis in the ER.

## Highlights

• Stress induces rapid ER export and degradation of misfolded GPI-anchored proteins
• ER export of misfolded GPI-anchored proteins relies on Tmp21
• Misfolded GPI-anchored proteins transit the cell surface en route to lysosomes
• ER export of misfolded GPI-anchored proteins prevents aggregation in ER during stress

## Graphical Abstract

**Figure:**
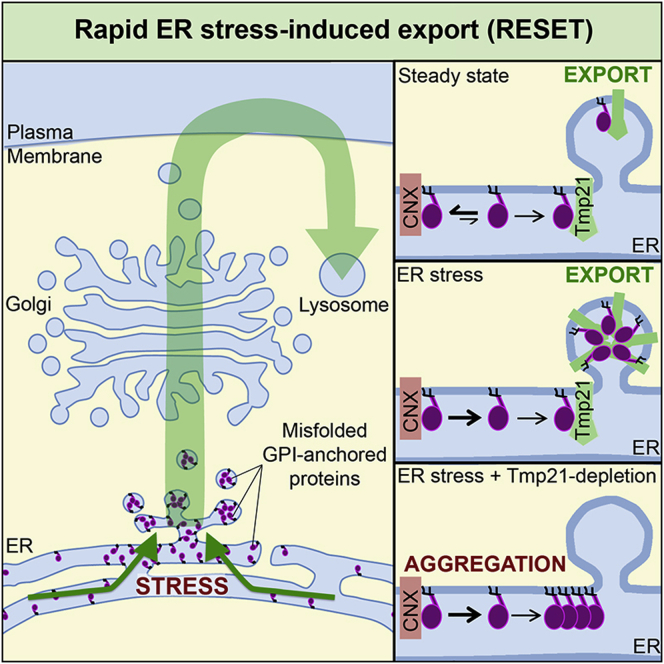

## Teaser

A previously unappreciated protective mechanism to alleviate acute ER stress has been identified. This ER stress-induced pathway triggers the export of misfolded GPI-anchored proteins, including disease-related prion mutants, for subsequent degradation in lysosomes.

## Introduction

Newly synthesized secretory and membrane proteins that do not pass quality control at the endoplasmic reticulum (ER) are typically retained by resident chaperones and routed to ER-associated degradation (ERAD) pathways (Hegde and Ploegh, 2010). Under some conditions, the burden of nascent unfolded and misfolded proteins in the ER increases beyond its processing capacity, leading to ER stress (Schröder and Kaufman, 2005). This activates the unfolded protein response (UPR), a multipronged signaling pathway that transcriptionally upregulates factors involved in expanding the ER protein folding capacity (Ron and Walter, 2007).

Although the UPR can restore protein folding homeostasis, the temporal lag of the transcriptional response (typically hours) raises the question of how protein quality control is achieved for misfolded proteins present in the ER during the acute phase of ER stress. Although the simplest answer is degradation by ERAD, these pathways would likely be temporarily saturated. Furthermore, recent work on mammalian prion protein (PrP) has suggested that at least some misfolded proteins may not be good substrates for ERAD.

PrP is a widely expressed cell surface glycosylphosphatidylinositol (GPI) anchored protein. Although the normal function of PrP is uncertain, its misfolding is causative of various diseases (Aguzzi et al., 2008; Prusiner, 1998). Among these, numerous natural and artificial misfolding or mislocalization mutants lead to neurodegeneration in both mice and humans (Kovács et al., 2002). Despite the importance of PrP misfolding in disease, the various pathways of misfolded PrP degradation are not well established.

Intriguingly, many PrP mutants that enter the ER lumen were found to be poorly degraded by ERAD, apparently relying instead on lysosomes (Ashok and Hegde, 2009). A notable exception was the situation in which addition of PrP’s GPI-anchor was blocked by either mutation or genetic perturbation, in which case the unprocessed PrP was routed efficiently for ERAD (Ashok and Hegde, 2008). These observations hinted at the possibility that GPI-anchored misfolded PrP was degraded by an undefined non-ERAD route. Such a pathway might be especially important during ER stress, a frequently encountered condition in vivo, including during PrP-induced neurodegeneration (Hetz and Soto, 2006). These considerations motivated us to investigate the fate of misfolded PrP along with other unrelated misfolded GPI-anchored proteins during acute ER stress.

Our experiments led us to a heretofore unappreciated pathway that clears a diverse range of misfolded GPI-anchored proteins from the ER within minutes of ER stress. These misfolded proteins synchronously enter the secretory pathway and briefly transit the plasma membrane before their final targeting to lysosomes for destruction. Knockdown of the major ER export factor, Tmp21, prevents this stress-induced egress, resulting in misfolded protein aggregation in the ER. The wide conservation of the GPI anchor in all eukaryotes and the fact that mammals express more than 150 different GPI-anchored proteins of critical function (Fujita and Kinoshita, 2012) highlight the importance of our findings.

## Results

### Stress-Induced Clearance of ER-Retained Misfolded PrP for Lysosomal Degradation

**Figure 1:**
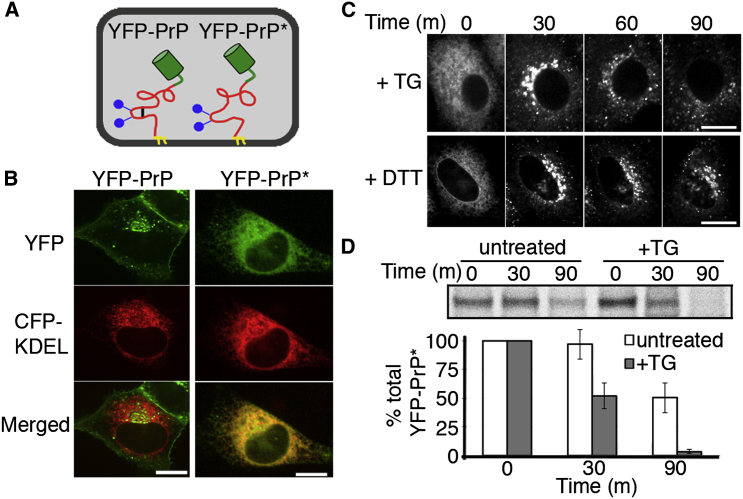
ER Stress Induces Rapid Relocalization and Degradation of ER-Retained Misfolded PrP (A) Diagrams of YFP-tagged wild-type PrP (YFP-PrP) and YFP-tagged misfolded PrP (YFP-PrP^∗^) depicting the GPI-anchor (yellow), two N-linked glycans (blue), the disulfide bond (black), and the YFP-tag (green). YFP-PrP^∗^ lacks the intramolecular disulfide bond. (B) Steady-state localization of YFP-PrP (left) and YFP-PrP^∗^ (right). CFP-KDEL marks the ER. (C) Time-lapse images of YFP-PrP^∗^-expressing cells after treatment with thapsigargin (TG, top) or with dithiothreitol (DTT, bottom) (D) Steady-state chase experiment performed using YFP-PrP^∗^-expressing cells. The top panel shows an autoradiograph of YFP-PrP^∗^ immunoprecipitations from a representative experiment, and the bottom panel shows quantification from multiple experiments (mean ± SE; n = 4). Scale bars, 10 μm. See also Movies S1 and S2 and Figure S1.

**Figure S1:**
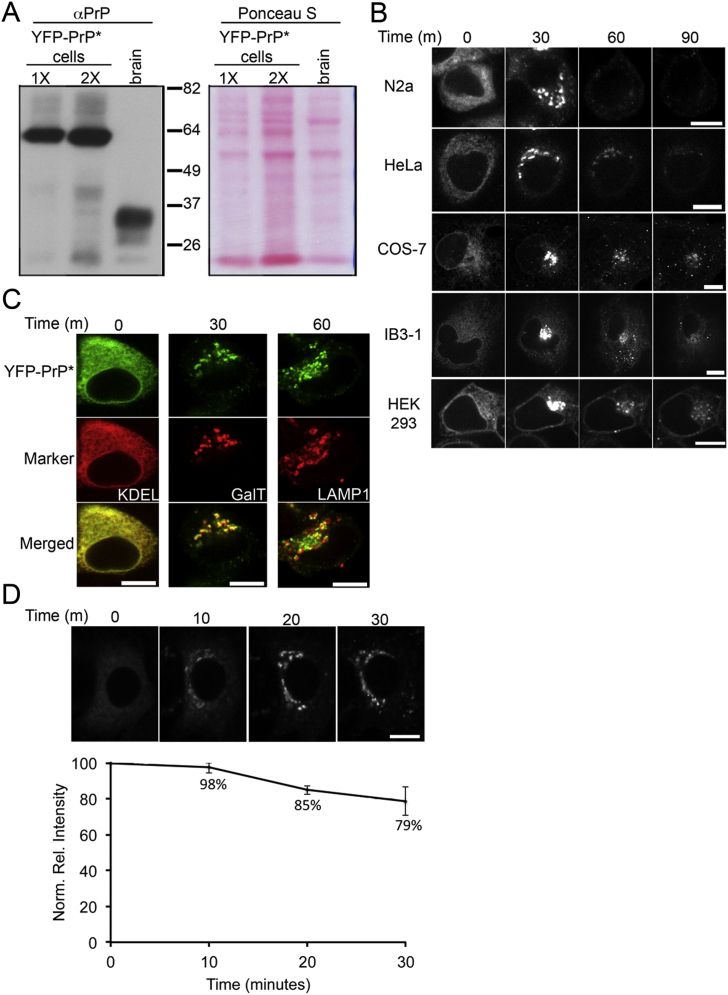
Characterization of YFP-PrP^∗^ Stress-Induced Degradation, Related to Figure 1 (A) Immunoblot for PrP (using the PrP-A antibody) comparing two different amounts (1X and 2X) of NRK cell lysate stably expressing YFP-PrP^∗^ with mouse brain homogenate. YFP-PrP^∗^ appears as a single ∼60 kDa band while endogenous mouse brain PrP appears as a set of ∼28-37 kDa bands. Ponceau S staining of the blot (right panel) reveals that the relative total protein concentration of YFP-PrP^∗^ cell lysate in the 1X loading lane is similar to mouse brain lysate. (B) The indicated cell lines were transiently transfected with YFP-PrP^∗^, and 48 hr later, treated with 0.1 μM thapsigargin (TG). Shown are images at different times after TG addition. Cells analyzed are mouse neuroblastoma (N2a), human cervical cancer (HeLa), African green monkey kidney (COS-7), bronchial epithelial (IB3-1), and human embryonic kidney (HEK293). (C) N2a cells were co-transfected with YFP-PrP^∗^ and the indicated organelle markers and, 48 hr later, treated with TG. Representative images of cells at different time points after TG treatment are shown. CFP-KDEL (KDEL) marks the ER. Resident Golgi protein galactosyltransferase T-CFP (GalT) marks the Golgi. LAMP1-CFP (LAMP1) marks the lysosomes. For the 60 min time point only, 125 μM leupeptin was used to inhibit lysosomal degradation so that YFP-PrP^∗^ could be visualized inside lysosomes. (D) Comparison of total YFP-PrP^∗^ fluorescence levels before and after stress-induced relocalization to post-ER compartments. Cells stably expressing YFP-PrP^∗^ were treated with TG and imaged at 10 min increments within the dynamic range of the camera. Images of a representative cell are shown above a graph quantifying 5 cells (mean ± standard error). By 30 min after acute ER stress, when all the detectable YFP-PrP^∗^ has left the ER and entered the secretory pathway, there is at least 79% of total starting fluorescence. Scale bars, 10 μm

We created a constitutively misfolding variant of PrP, hereafter named PrP^∗^, by perturbing the essential disulfide bond between cysteine residues 179 and 214 with a C179A point mutation (Figure 1A). PrP^∗^ was fluorescent protein (FP) tagged at a site previously shown not to disrupt wild-type PrP folding or trafficking (Ashok and Hegde, 2009). Our construct was equipped with the prolactin signal sequence to ensure efficient translocation into the ER (Kang et al., 2006) and stably expressed in cultured cells at physiological levels (Figure S1A available online). In contrast to cell-surface localized wild-type YFP-PrP, constitutively misfolded YFP-PrP^∗^ was almost entirely retained in the ER at steady state in multiple cell types (Figures 1B, S1B, and S1C).

Upon application of two independent ER stressors, thapsigargin (TG) and dithiothreitol (DTT), live-cell time-lapse imaging revealed rapid relocalization of YFP-PrP^∗^ from the ER to a perinuclear locale (within 10–30 min), distributed puncta (within 30–60 min), and disappearance from those puncta (by 60–120 min) (Figure 1C and Movies S1 and S2). Biochemical analysis of radiolabeled YFP-PrP^∗^ verified that acute ER stress accelerates its degradation relative to untreated cells (Figure 1D). As we discovered later (see below), untreated cells also employ the same degradation pathway at low levels, explaining why∼50% of YFP-PrP^∗^ is lost by 90 min. Similar stress-enhanced degradation was observed for other disulfide mutants of PrP, including C179S, C214A, or C214S (data not shown) and human disease mutants of PrP (described below). Thus, misfolded PrPs residing in the ER at the onset of acute ER stress are rapidly degraded by a pathway involving its relocalization.

**Figure 2:**
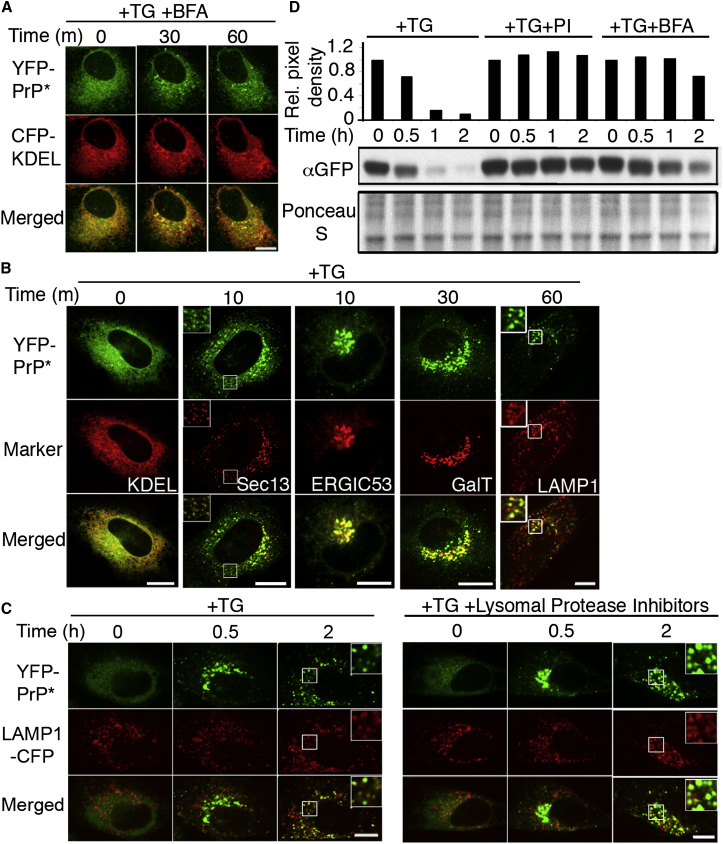
PrP^∗^ Undergoes Rapid ER Stress-Induced Export for Subsequent Lysosomal Degradation (A) Cells cotransfected with CFP-KDEL and YFP-PrP^∗^ were treated with brefeldin A (BFA) and TG, and time-lapse images were collected. (B) Cells were cotransfected with YFP-PrP^∗^ and a CFP-tagged fusion of the indicated organelle marker. Images of different cells were collected at the indicated times after TG treatment. KDEL, Sec13, ERGIC53, GalT, and LAMP1 each respectively mark the ER, ER exit sites, ER-Golgi intermediate compartment, Golgi, and lysosomes. (C) YFP-PrP^∗^ and LAMP1-CFP-expressing cells were treated with TG or TG plus lysosomal protease inhibitors, and time-lapse images were taken. The inset at the 2 hr time point shows a magnified view of the region marked by the box. Complete data set in Figure S2B. (D) Representative western blot (middle) and quantification (top) from lysates of YFP-PrP^∗^-expressing cells that were either treated with TG alone (+TG), TG plus protease inhibitors (TG+PI), or TG plus BFA. Densitometry of YFP-PrP^∗^ was normalized using the Ponceau S stained blot (bottom). Scale bars, 10 μm. See also Figures S1 and S2.

An inhibitor of secretory traffic, brefeldin A (BFA) (Klausner et al., 1992), blocked the ER-stress-induced relocalization of PrP^∗^, suggesting that ER export was involved (Figure 2A). Colocalization experiments at different times after stress induction showed that YFP-PrP^∗^ transits successively through the ER, ER exit sites, ER-Golgi intermediate compartment, Golgi, and lysosomes (Figure 2B). YFP-PrP^∗^ followed the same itinerary in multiple cell types derived from different tissues (Figures S1B and S1C).

**Figure S2:**
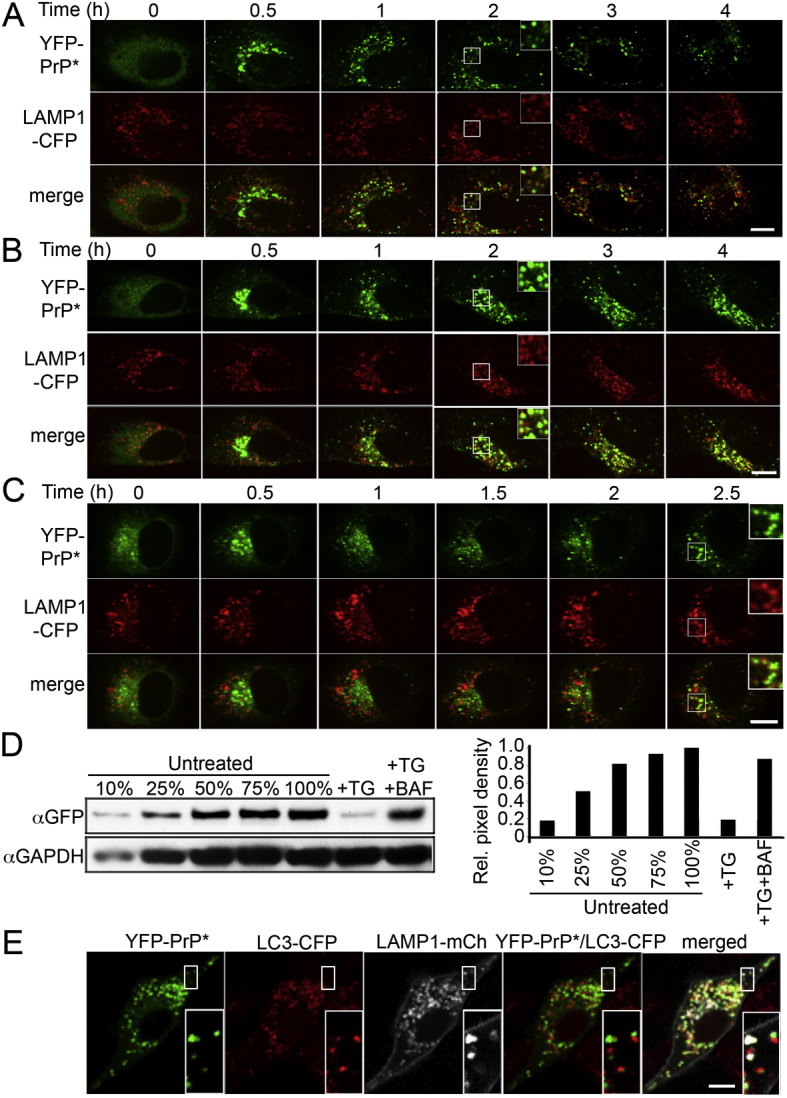
Lysosomal Degradation of YFP-PrP^∗^, Related to Figure 2 (A–C) Forty-eight hours after transfection with YFP-PrP^∗^ and LAMP1-CFP, NRK cells were treated as follows and time-lapse movies were collected: (A) 0.1 μM thapsigargin (TG), (B) TG with lysosomal protease inhibitors (125 μM leupeptin + protease inhibitor cocktail (Sigma P8340) diluted 1:100), or (C) TG with 250nM bafilomycin A1. Note that bafilomycin A1 hampers efflux through the Golgi relative to TG alone or TG with leupeptin. However YFP-PrP^∗^ ultimately accumulates in lysosomes by 2 hr after TG + BAF treatment. The insets show a magnified view of the region marked by the box. (D) NRK cells stably expressing YFP-PrP^∗^ were treated with 0.1 μm thapsigargin (TG) or TG plus 250 nM bafilomycin A1 for 2 hr after a 30 min bafilomycin pretreatment (TG+BAF). The lysates were analyzed by immunoblotting for YFP-PrP^∗^ and GAPDH relative to serial dilutions of untreated lysate for quantification (right panel). E, NRK cells stably expressing the autophagosome marker LC3-CFP were co-transfected with LAMP1-mCherry and YFP-PrP^∗^. Forty-eight hours after transfection, cells were treated with 250nM bafilomycin A1 for 6 hr and an image was collected. Bafilomycin A1 inhibits autophagosome-lysosome fusion in addition to inhibiting lysosomal degradation, allowing YFP-PrP^∗^ localization to be determined. The insets show a magnified view of the region marked by the box. Scale bars, 10 μm

Quantitative microscopy of the trafficking pathway that YFP-PrP^∗^ followed during ER stress revealed that more than 80% of the protein resided solely in post-ER compartments prior to being degraded (Figure S1D). This suggests that ERAD pathways are not a major determinant of PrP^∗^ degradation during ER stress. Instead, its lysosomal localization prior to disappearance indicated that lysosomes are the major site of PrP^∗^ degradation (Figures 2B and 2C). Interfering with lysosomal function by inhibiting either its resident proteases or its acidification (with bafilomycin A1) increased PrP^∗^ accumulation in lysosomes (Figures 2C and S2A–2C). Immunoblotting analysis verified that full-length YFP-PrP^∗^ was indeed stabilized with BFA, lysosomal inhibitor, or bafilomycin A1 treatments (Figures 2D and S2D).

Of note, we determined that autophagy does not play a role in relocalization of YFP-PrP^∗^ to lysosomes by using bafilomycin A1, which blocks autophagosome fusion with lysosomes in addition to inhibiting lysosomal degradation (Yamamoto et al., 1998). YFP-PrP^∗^ accumulated within lysosomes rather than in autophagosomes under these conditions (Figure S2E). Furthermore, inhibition of autophagy with 3-methyladenine had no effect on PrP^∗^ relocalization to lysosomes (data not shown).

These results reveal a stress-induced process for export of misfolded ER-retained proteins to downstream compartments of the secretory pathway for subsequent lysosomal degradation. We term this ER-clearance process RESET (for *r*apid *E*R *s*tress-induced *e*xpor*t*) to distinguish it from qualitatively different ER-clearance pathways that involve ERAD or autophagy.

### Misfolded PrP Accesses the Cell Surface during Transit to Lysosomes

**Figure 3:**
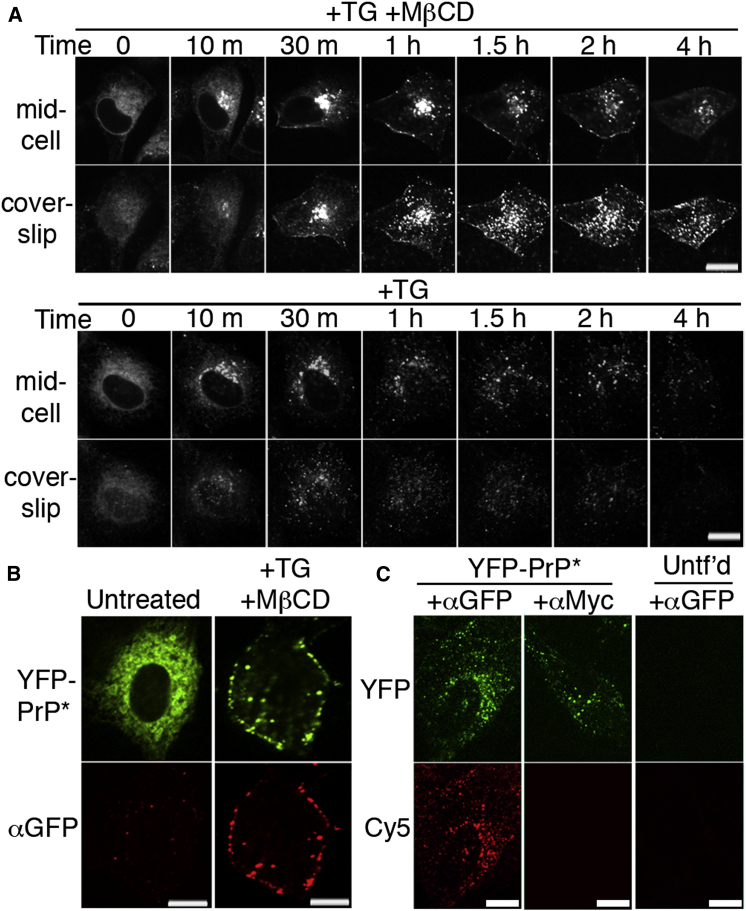
PrP^∗^ Traffics to the Plasma Membrane during RESET (A) Time-lapse images of YFP-PrP^∗^-expressing cells treated with TG + methyl-β-cyclodextrin (MβCD) (top) or TG alone (bottom) taken at two different focal planes. Mid-cell indicates a focal plane at the widest point of the nucleus and coverslip indicates a focal plane close to the coverslip where the largest portion of the plasma membrane is in focus. (B) YFP-PrP^∗^ cells were untreated or treated with TG and MβCD and then fixed after 90 min and stained with anti-GFP antibody without permeabilization to detect cell surface YFP-PrP^∗^. (C) Antibody uptake assay for internalization of YFP-PrP^∗^ from the plasma membrane. YFP-PrP^∗^-expressing or untransfected (Untf’d) cells were treated for 1 hr with TG in the presence of leupeptin and either anti-GFP or anti-Myc. The cells were then washed, fixed, permeabilized, and stained with Cy5-conjugated secondary antibody to detect internalized antibody. Scale bars, 10 μm.

Trafficking to lysosomes can either be direct from the Golgi or indirect via the plasma membrane (Arvan et al., 2002; Luzio et al., 2003). To see whether YFP-PrP^∗^ accesses the cell surface en route to lysosomes, we inhibited the major endocytosis pathways with the cholesterol-depleting agent methyl-β-cyclodextrin (MβCD) (Kirkham and Parton, 2005; Rodal et al., 1999). When YFP-PrP^∗^ molecules were released from the ER with stress in the presence of MβCD, a substantial proportion of these molecules was observed to be trapped at the plasma membrane (Figure 3A) and accessible to extracellular antibody (Figure 3B). Antibody uptake assays in the absence of MβCD verified that stress-released YFP-PrP^∗^ that ultimately arrived in lysosomes had sampled the cell surface (Figure 3C). Its apparent short residence time at the plasma membrane relative to wild-type PrP (Figure 1B) implicates poorly understood quality control pathways at the plasma membrane for selective internalization and destruction in lysosomes.

### Diverse Misfolded GPI-Anchored Proteins Access the RESET Pathway

**Figure 4:**
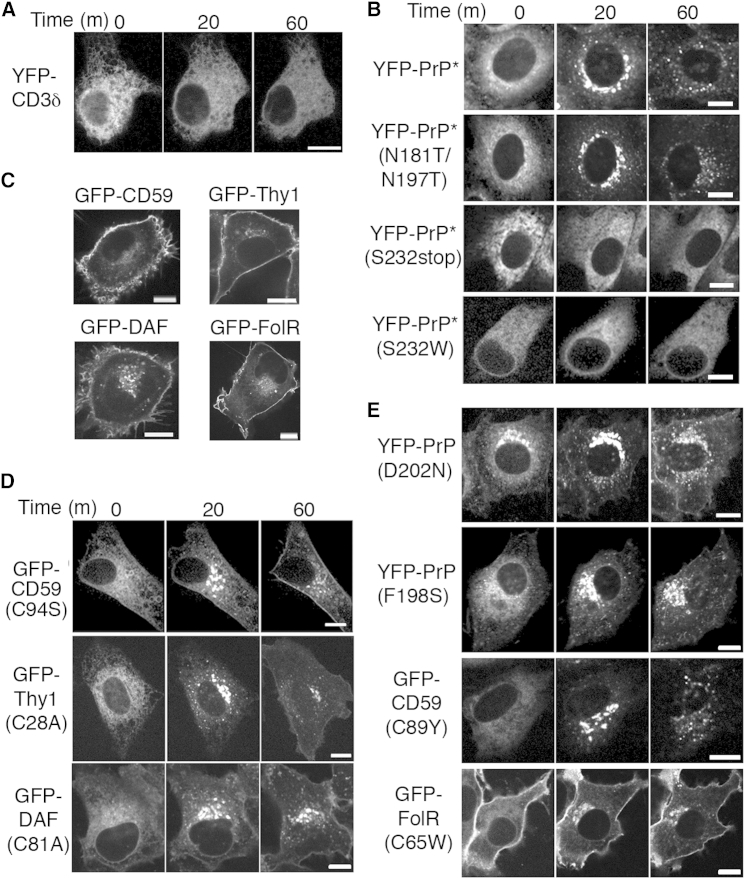
Diverse GPI-Anchored Proteins Undergo ER Stress-Induced Export (A) Time-lapse images captured after addition of TG to cells stably expressing the ERAD substrate YFP-CD3δ. (B) Time-lapse images after TG addition to cells expressing the indicated YFP-PrP^∗^ mutants. N181T/N197T prevents both N-glycosylations, S232stop truncates the GPI-anchor signal sequence, and S232W blocks processing of the GPI-anchor signal sequence. (C) Steady-state localization of the indicated GFP-tagged wild-type GPI-anchored proteins. (D and E) Time-lapse images after TG addition to cells expressing the indicated mutants of GPI-anchored proteins. Experiments were performed 48 hr after transfection. (D) Artificial mutants include GFP-tagged CD59 (C94S, to disrupt one of five potential internal disulfide bonds), Thy1 (C28A, to disrupt one of three potential internal disulfide bonds), and decay accelerating factor (DAF, C81A, to disrupt one of eight potential internal disulfide bonds). (E) Naturally occurring human disease mutants include PrP (D202N), PrP (F198S), CD59 (C89Y), and FolR (C65W). Scale bars, 10 μm. See also Figure S3.

**Figure S3:**
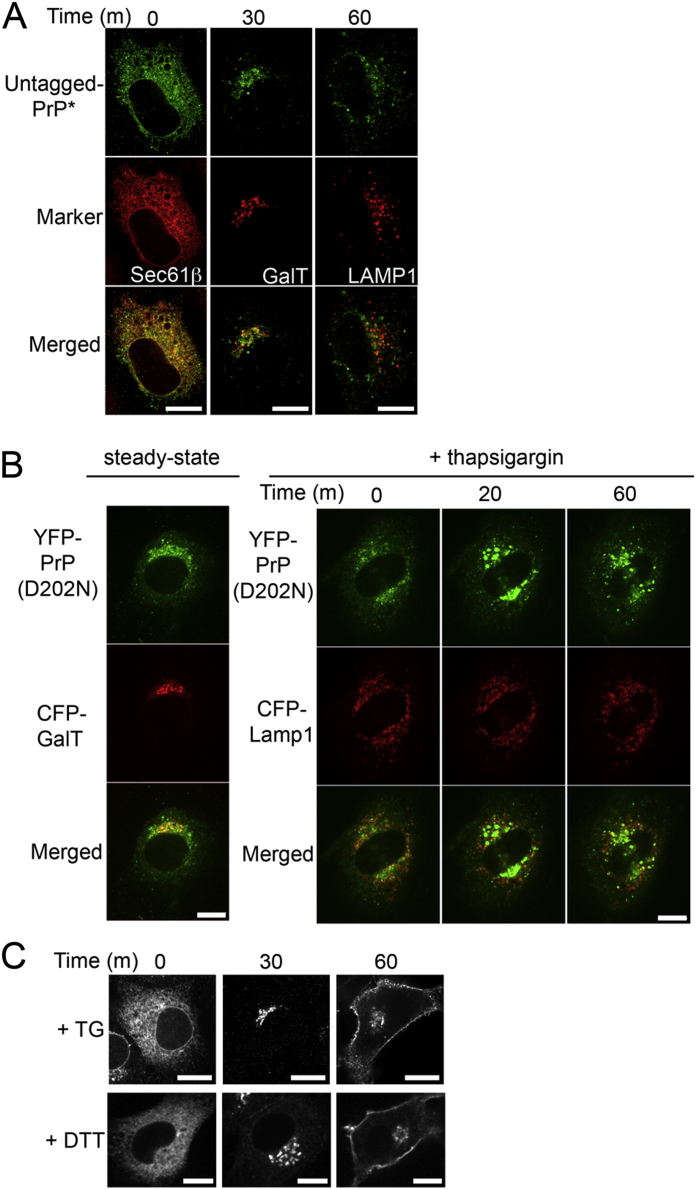
Generality of the RESET Pathway, Related to Figure 4 (A) NRK cells were co-transfected with untagged-PrP^∗^ and a single organelle marker approximately 48 hr prior to treatment with 0.1 μM thapsigargin (TG). At the indicated times after TG-treatment, cells were fixed and immunostained with PrP-A antibody to detect the untagged-PrP^∗^. For each panel the individual channels and merged image are shown. The ER is marked with CFP-Sec61β. The Golgi is marked by galactosyltransferase T-CFP (GalT). Lysosomes are marked by LAMP1-CFP (LAMP1). The cells marked by LAMP1-CFP were also treated with 125 μM leupeptin to inhibit lysosomal degradation of PrP^∗^. (B) NRK cells co-transfected with the human disease mutant YFP-PrP(D202N) and the indicated organelle marker were imaged at steady state (left) or at different times after TG-treatment (right panel). (C), NRK cells transfected for 48 hr with TCR-α_GPI_ were treated with either TG or DTT for the indicated times before fixation and immunofluorescent detection of TCR-α. Scale bars, 10 μm

The ER-retained T cell receptor subunit, CD3δ, was not exported during acute ER stress (Figure 4A) and instead was degraded by ERAD (Klausner et al., 1990). This suggested that RESET may be dependent on a feature of the ER quality control substrate. Domain analysis of YFP-PrP^∗^ revealed that one element regulating its RESET is the GPI anchor. Either preventing GPI-anchor addition (with the processing site mutation S232W [Ashok and Hegde, 2008]) or deleting the GPI-anchoring signal resulted in an ER-retained misfolded protein that was not exported to downstream compartments of the secretory pathway during stress (Figure 4B). By contrast, RESET was unaffected by preventing N-linked glycosylation of YFP-PrP^∗^ (with mutations to both asparagines, N181T and N197T) (Figure 4B) or eliminating the YFP-tag (Figure S3A).

We tested whether the GPI anchor may be a general signal to route misfolded proteins for RESET by examining artificial and naturally occurring mutants of GPI-anchored proteins whose GFP-tagged wild-type versions traffic normally to the plasma membrane (Figure 4C). The ER-retained pool of disulfide mutants of three unrelated GPI-anchored proteins—CD59, Thy1, and DAF—underwent RESET (Figure 4D). Importantly, RESET was not limited to artificial mutants but also was seen with several human disease-associated mutants (Figures 4E and S3B). These include the D202N and F198S mutants in PrP (Gu et al., 2007; Ivanova et al., 2001), the C89Y mutant in CD59 (Nevo et al., 2013), and the C65W mutant in folate receptor 1 (Grapp et al., 2012). These results suggest that GPI-anchored proteins represent a major client set for RESET.

This conclusion is consistent with old observations on the fate of the transmembrane TCR-α, an unassembled T cell receptor subunit and ERAD substrate (Suzuki et al., 1991). Although a soluble truncated version of TCR-α lacking the cytosolic and transmembrane domains (TCR-α_t_) was found to be retained in the ER in thapsigargin-treated cells, a variant in which TCR-α_τ_ is appended to a GPI-anchor (TCR-α_GPI_) is exported to the cell surface. We verified this result and found that RESET of TCR-α_GPI_ is seen with either thapsigargin- or DTT-mediated ER stress (Figure S3C). Thus, an ERAD substrate can be converted to a RESET substrate solely by the addition of a GPI-anchor, identifying this as at least one major feature of a RESET client.

### RESET Clients Utilize Tmp21-Mediated ER Export

**Figure S4:**
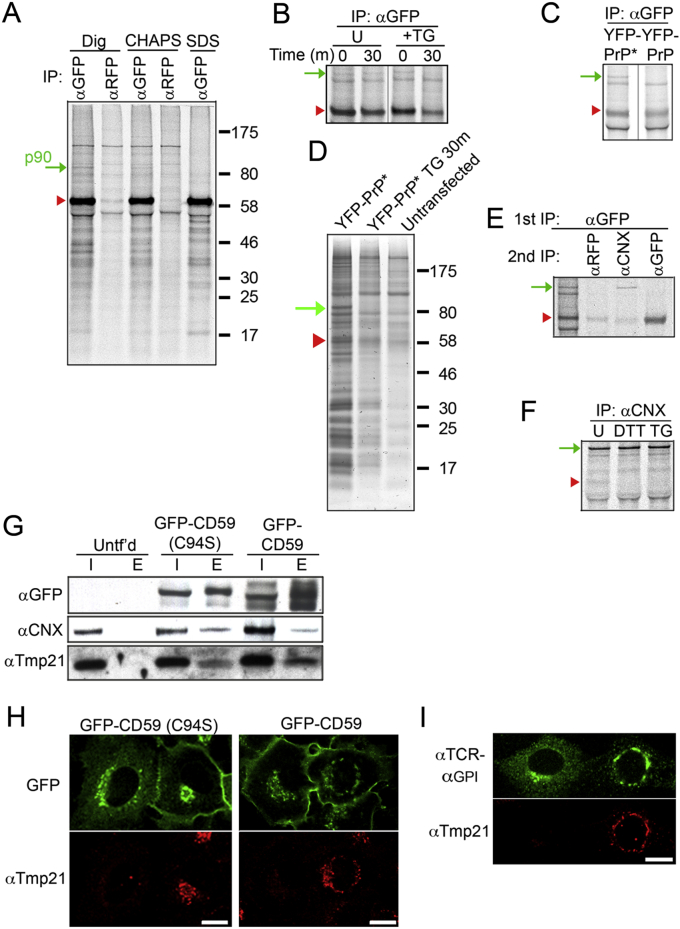
Retention and Release of GPI-Anchored Proteins during RESET, Related to Figure 5 (A) Radiolabeled NRK cells stably expressing YFP-PrP^∗^ were lysed under denaturing conditions using 1% SDS or non-denaturing conditions using 1% digitonin (Dig) or 1% CHAPS. The lysates were subjected to immunoprecipitation using anti-GFP or anti-RFP (negative control) and analyzed by SDS-PAGE and autoradiography. The red arrowhead marks the position of YFP-PrP^∗^ and the green arrow marks the position of a ∼90 KDa associated protein (p90) only observed under non-denaturing conditions specifically in the GFP-IPs. (B) An experiment similar to panel A (using 1% CHAPS) on cells that were untreated or treated with 0.1 μM thapsigargin (TG) for 30 min. The co-precipitating p90 band disappears within 30 min of TG treatment. (C) An experiment similar to panel A (using CHAPS) comparing the co-IP profiles of YFP-PrP^∗^ versus YFP-PrP retained in the ER with 1 μM brefeldin A (BFA). The p90 band is selective to YFP-PrP^∗^. (D) GFP pulldowns from, YFP-PrP^∗^ stably expressing cells that were either untreated or treated TG for 30 min. Untransfected cells were included as a negative control. The eluates were analyzed by Coomassie staining, and the p90 band was identified as calnexin by mass spectrometry. (E) A co-IP sample similar to the YFP-PrP^∗^ lane of panel C was denatured and either analyzed directly (first lane) or re-immunoprecipitated using the indicated antibodies. p90 was confirmed to be calnexin. (F) An experiment similar to panel B was performed, but with anti-CNX immunoprecipitation. The YFP-PrP^∗^ product disappears upon DTT or TG treatment. (G) Lysates of the indicated cells were subjected to GFP pulldowns and the input (I) and eluate (E) fractions analyzed by Western blot with antibodies against GFP, CNX, and Tmp21. The cells were either untransfected (Untf’d), stably expressing GFP-CD59 (C94S), or stably expressing GFP-CD59 treated with 1 μM BFA for 3 hr to retain GFP-CD59 in the ER. (H) NRK cells stably expressing GFP-CD59 or GFP-CD59 (C94S) were treated with Tmp21-siRNA and analyzed for localization of GFP and Tmp21 immunofluorescence. The representative panel shows two neighboring cells where the cell on the left had significant Tmp21 knockdown, while the cell on the right did not. In all cases, lack of Tmp21 staining coincided with inhibition of export of the ER-retained population of mutant GFP-CD59 (n = 103), while the presence of Tmp21 coincided with mutant GFP-CD59 localization to the Golgi and cell surface (n = 97). Wild-type GFP-CD59 cells were treated with BFA for 3 hr to retain newly synthesized protein in the ER, released from the BFA block for 60 min, then fixed and analyzed. In all cases, GFP-CD59 was exported from ER and was detected at the plasma membrane regardless of Tmp21 knockdown. (I) NRK cells were transfected with TCRα-GPI, treated with Tmp21-siRNA, and analyzed similarly to panel H. In all cases, lack of Tmp21 staining coincided with inhibition of export of the ER-retained population of TCRα-GPI (n = 36), while detection of Tmp21 coincided with TCRα-GPI localization to the Golgi (n = 64). Scale bars, 10 μm

We next determined the mechanistic basis of RESET. RESET presumably operates by switching from retention of the misfolded protein in the ER under nonstressed conditions to egress during ER stress. This implies the existence of a retention factor and an export receptor. A candidate retention factor for YFP-PrP^∗^ was identified by immunoprecipitation and mass spectrometry. In these experiments, we looked for ER-resident interaction partners that release very shortly after application of ER stressors. An ∼90 kD band first detected in radiolabeled coimmunoprecipitation experiments (Figures S4A–S4F) was identified by mass spectrometry as calnexin, an ER-resident chaperone (Bergeron et al., 1994). The reliance on a GPI-anchor for RESET of YFP-PrP^∗^ implicated the p24 family of export receptors for GPI-anchored protein trafficking (Bonnon et al., 2010; Schimmöller et al., 1995; Takida et al., 2008). We therefore scrutinized these factors for their potential role in RESET.

**Figure 5:**
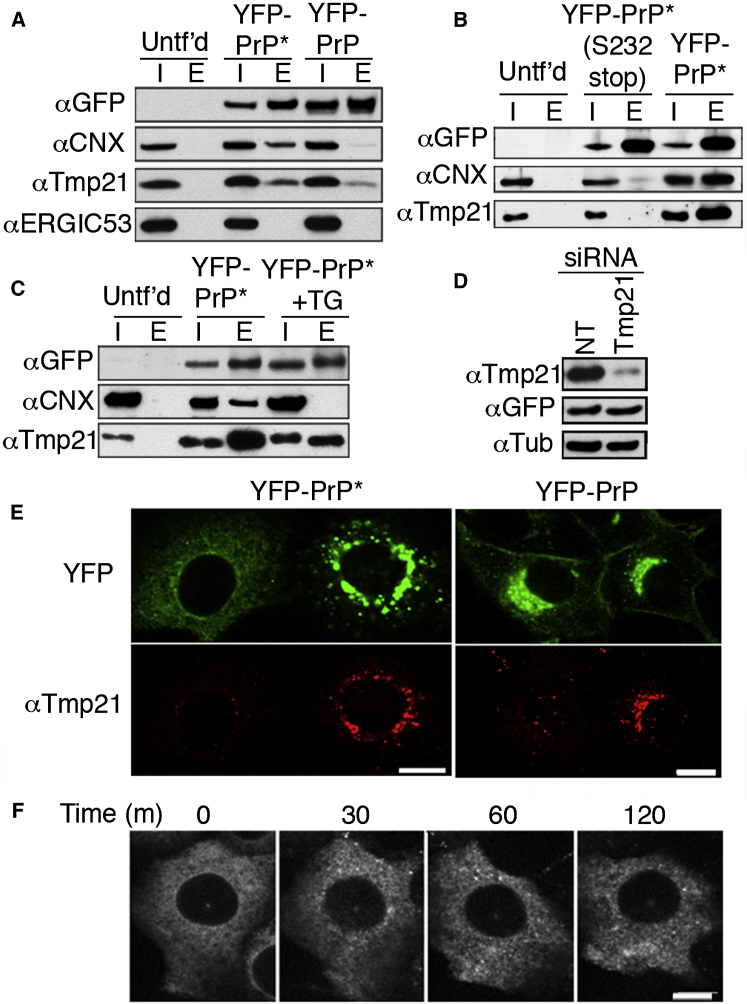
PrP^∗^ Releases from Calnexin upon ER Stress for Tmp21-Dependent Export (A–C) Cells stably expressing the indicated PrP constructs were subjected to GFP pull-downs and input (I) and eluate (E) fractions analyzed by western blot for the indicated proteins, including calnexin (CNX), Tmp21, and p58/ERGIC53. Untransfected cells (Untf’d) served as a negative control. In (A), YFP-PrP-expressing cells were treated with BFA for 3 hr to retain newly synthesized YFP-PrP in the ER. In (C), TG treatment, where indicated, was for 30 min. (D) Western blot of YFP-PrP^∗^ cells treated with nontargeting (NT) or Tmp21 siRNA probed for Tmp21, YFP-PrP^∗^, or tubulin. (E) Cells expressing either YFP-PrP^∗^ (left) or YFP-PrP (right) were subjected to siRNA treatment against Tmp21 and analyzed for YFP localization and Tmp21 immunofluorescence. Each panel shows two adjacent cells in the same field of view where one cell was depleted for Tmp21 and the other cell was not. YFP-PrP^∗^ cells were treated with TG for 30 min prior to fixation, whereas YFP-PrP cells were treated with BFA for 3 hr, released for 60 min, and then fixed. Quantification of multiple fields showed that YFP-PrP^∗^ was ER localized in all Tmp21-depleted cells (n = 133), but Golgi localized in Tmp21-expressing cells (n = 117). By contrast, YFP-PrP was detected in vesicular compartments and the plasma membrane regardless of Tmp21 knockdown (n = 200). (F) Time-lapse images after TG treatment of YFP-PrP^∗^ in a cell knocked down for Tmp21. Identical results were obtained in ∼50% of all cells (n = 112), which is consistent with the proportion of cells successfully knocked down for Tmp21. Scale bars, 10 μm. See also Figure S4.

Coimmunoprecipitation experiments showed that calnexin associates with YFP-PrP^∗^, but not as strongly with wild-type YFP-PrP, suggesting a misfolding-dependent interaction (Figures 5A and S4C). The p24 family member, Tmp21, interacted with both misfolded and wild-type PrPs (Figure 5A). By contrast, the unrelated ER export receptor ERGIC53 showed no interaction with either YFP-PrP^∗^ or YFP-PrP, providing a specificity control. The interactions with Tmp21 and calnexin were strongly dependent on the GPI anchor (Figure 5B), which is consistent with their potential role in RESET.

Upon induction of ER stress, the interaction with calnexin was lost, whereas the Tmp21 interaction was maintained (Figure 5C). YFP-PrP^∗^ egress proved to be dependent on Tmp21, as evidenced by a strong failure in RESET in Tmp21 knockdown cells (Figures 5D–5F). Similarly, calnexin and Tmp21 interaction were observed for misfolded CD59 (Figure S4G), and dependence on Tmp21 for stress-induced ER export was shown for CD59 (Figure S4H) and TCR-α_GPI_ (Figure S4I). Collectively, these results demonstrate that Tmp21 plays a key role in the RESET of misfolded GPI-anchored proteins.

Notably, wild-type PrP and CD59 showed no gross defects in their steady-state plasma membrane localization in Tmp21 knockdown cells (Figures 5D and S4H). This is consistent with an earlier study that showed only a mild kinetic delay in wild-type GPI-anchored protein trafficking in the absence of Tmp21 (Takida et al., 2008). The far stronger dependence on Tmp21 for misfolded GPI-anchored protein ER export implicates a role for Tmp21 in maintenance of ER protein folding homeostasis.

### ER Stress Drives Aggregation of Misfolded PrP in the ER of Tmp21-Depleted Cells

**Figure 6:**
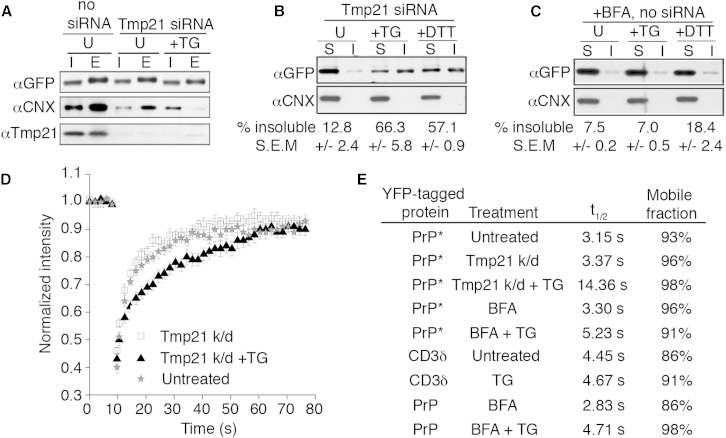
PrP^∗^ Aggregates in the ER upon Selective Inhibition of RESET (A) YFP-PrP^∗^-expressing cells were subjected to Tmp21 siRNA and treated with TG for 30 min where indicated and subjected to GFP pull-downs. The input (I) and eluate (E) fractions were analyzed by immunoblotting for the indicated proteins. (B) YFP-PrP^∗^-expressing cells subjected to Tmp21 siRNA were treated for 60 min with TG or DTT as indicated. Their cell lysates were separated into soluble (S) and insoluble (I) fractions and analyzed by western blot for YFP-PrP^∗^. The percent insoluble was calculated from three experiments and shown below the gel (mean ± SE). (C) YFP-PrP^∗^-expressing cells were treated with BFA, BFA plus TG, or BFA plus DTT for 60 min and analyzed as in (B). (D) FRAP analysis was performed on YFP-PrP^∗^ in untreated cells or cells depleted for Tmp21 (Tmp21 k/d) with or without 60 min of TG treatment. Average normalized fluorescence intensity values were fit to an exponential curve to determine recovery half-times (t_1/2_). Error bars indicate SE. (E) Table of half-times and mobile fractions compiling data from (C) and from Figures S5A–5C for comparison. See also Figure S5.

To clarify the physiologic role of RESET in maintaining ER homeostasis, we depleted Tmp21 and assessed the biochemical and biophysical properties of ER-retained PrP^∗^. YFP-PrP^∗^ in unstressed Tmp21 knockdown cells was associated with calnexin by coimmunoprecipitation (Figure 6A) and is predominantly soluble in nondenaturing detergents (Figure 6B). Upon ER stress, the calnexin interaction was lost (Figure 6A), despite the fact that YFP-PrP^∗^ does not exit the ER in Tmp21 knockdown cells (Figures 5D–5F). This loss of chaperone interaction was accompanied by YFP-PrP^∗^ becoming substantially detergent insoluble (Figure 6B), suggesting that it had partially aggregated.

In cells containing Tmp21, YPF-PrP^∗^ would exit the ER upon ER stress, thereby escaping aggregation in the ER. To test whether YFP-PrP^∗^ aggregation in Tmp21 knockdown cells was simply a consequence of preventing this exit, we analyzed control cells subjected to ER stress in the presence of BFA to prevent ER export (Figure 6C). Instead of aggregating, YFP-PrP^∗^ remained largely soluble under these conditions. This suggests that Tmp21, beyond simply exporting YFP-PrP^∗^ out of a stressed ER, plays a role in precluding its aggregation. Such a function for Tmp21 may be due to its direct interaction with YFP-PrP^∗^, although this remains to be examined in future studies.

**Figure S5:**
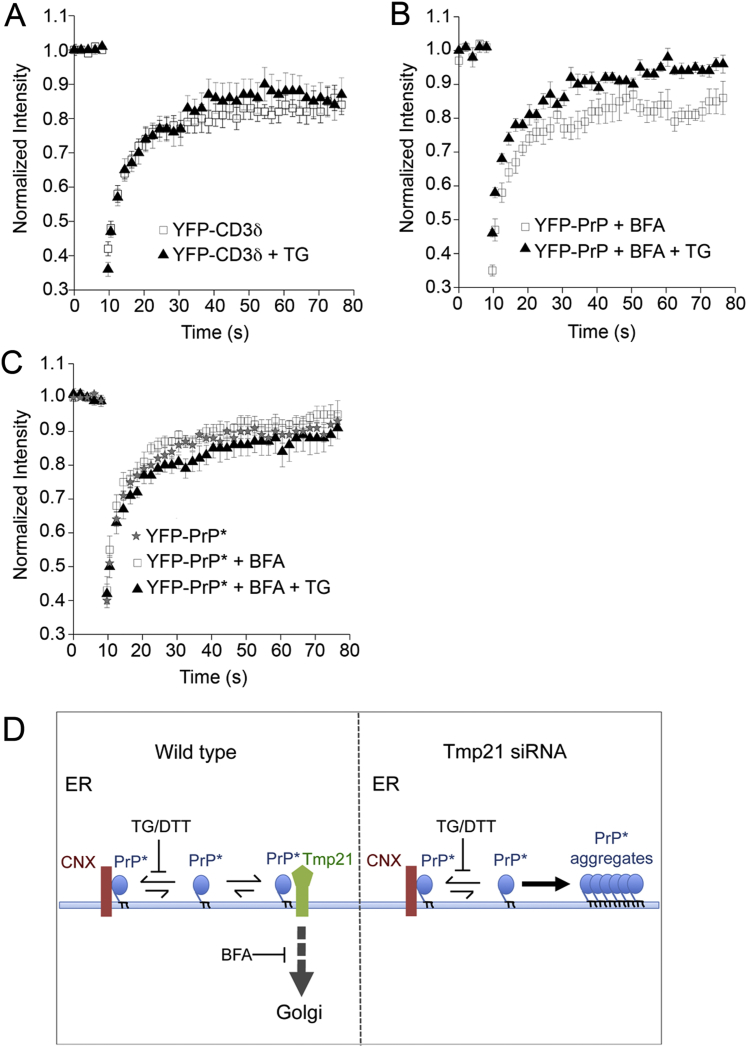
ER Stress Effects on Protein Mobility in the ER, Related to Figure 6 (A) Cells stably expressing the ER retained misfolded protein YFP-CD3δ were either left untreated or treated with TG for 30 min before analysis by fluorescence recovery after photobleaching (FRAP). The recovery curves are nearly identical. Error bars indicate standard error. (B) YFP-PrP was retained in the ER with 1 μM brefeldin A (BFA) for 3 hr and treated as indicated with TG for 30 min. The cells were then analyzed by FRAP. (C) YFP-PrP^∗^ expressing cells were either left untreated, or treated with 1 μM brefeldin A (BFA) with or without 60 min of 0.1 μM thapsigargin (TG)-treatment. They were then analyzed by FRAP. (D) Model explaining the results from the solubility assays and FRAP analyses shown here and in Figure 6. In wild-type cells (left), PrP^∗^ is predominantly maintained in a soluble state through its interaction with calnexin. Induction of acute ER stress with either TG or DTT limits calnexin availability for PrP^∗^ binding, allowing PrP^∗^ to bind Tmp21. Tmp21 maintains PrP^∗^ in a soluble state and facilitates PrP^∗^ export to the secretory pathway. Although ER export can be blocked using BFA, this does not lead to PrP^∗^ aggregation in cells where Tmp21 is available to bind PrP^∗^. In Tmp21-depleted cells (right), ER stress results in calnexin release; however, in the absence of Tmp21, PrP^∗^ can neither be exported nor shielded from aggregation.

In addition to the biochemical analysis, we studied the diffusion properties of YFP-PrP^∗^ in live cells using fluorescence recovery after photobleaching (FRAP) (Figures 6D and 6E). YFP-PrP^∗^ in the ER of unstressed cells, with or without Tmp21 knockdown, displayed rapid diffusion with a t_1/2_ of fluorescence recovery of ∼3 s. By contrast, YFP-PrP^∗^ in the stressed ER of Tmp21 knockdown cells had a nearly 5-fold slower recovery rate with a t_1/2_ of ∼14 s. Importantly, ER stress caused at most a modest change in mobility for YFP-CD3δ, YFP-PrP^∗^, or wild-type YFP-PrP retained in the ER by BFA (Figures 6E and S5A–S5C). Thus, a dramatic reduction of diffusion is selective to the RESET substrate PrP^∗^ and depends on the triad of ER stress, Tmp21 depletion, and PrP misfolding.

The results from the solubility assays and FRAP analyses are consistent with a model wherein Tmp21-mediated RESET is critical for avoiding PrP^∗^ aggregation in the ER during ER stress (Figure S5D). Accordingly, in wild-type cells, most PrP^∗^ is maintained in a soluble state through its interaction with ER chaperones, whereas under acute ER stress conditions, when chaperone availability is limiting, PrP^∗^ can bind to Tmp21. Tmp21-PrP^∗^ interaction fulfills two parallel functions: (1) maintaining PrP^∗^ in a soluble state and (2) facilitating PrP^∗^ export from the ER. In support of this model, Tmp21 depletion leads to PrP^∗^ retention in the ER and its aggregation in stressed cells, whereas in cells in which Tmp21 is available, PrP^∗^ that is retained in the ER by BFA treatment does not aggregate with stress.

### Relationship between RESET and the Unfolded Protein Response

To explore the relationship between RESET and the UPR, we generated a YFP-PrP^∗^-expressing cell line that was stably transfected with an XBP1-mCherry reporter for UPR induction (Brunsing et al., 2008). ER stress activates Ire1 to initiate the UPR by splicing XBP1 mRNA, allowing for expression of the XBP1 transcription factor (Yoshida et al., 2001). Therefore, we could simultaneously monitor in live cells the RESET of PrP^∗^ and the ER stress response, as revealed by XBP1-mCherry fluorescence.

**Figure 7:**
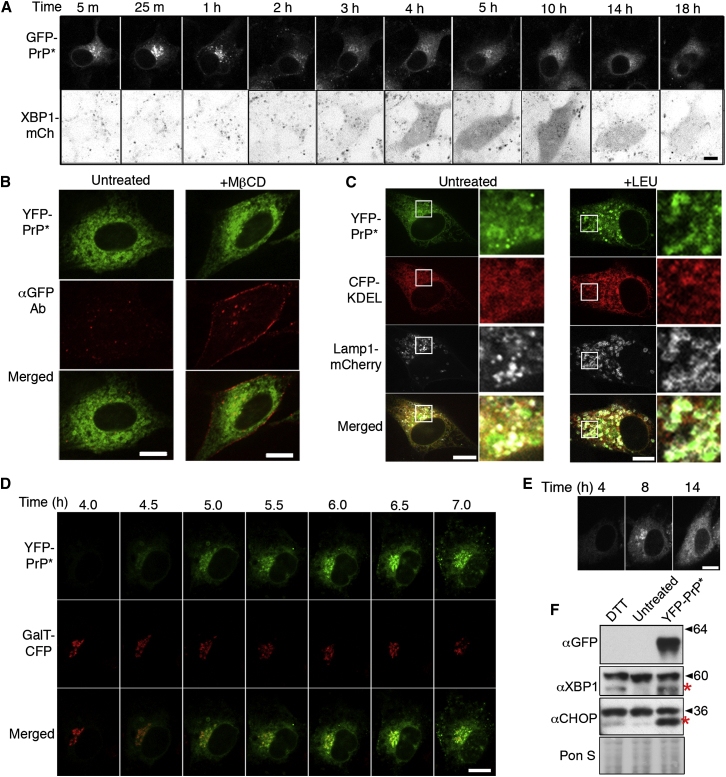
The ER Stress Response Modulates ER Export of YFP-PrP^∗^ (A) Cells stably expressing XBP1-mCherry were transiently transfected with GFP-PrP^∗^ and, 48 hr later, treated with DTT. Shown are time-lapse images over the course of 18 hr starting 5 min after addition of DTT. Extended time lapse series data are shown in Figure S6A. (B) Nonpermeabilized YFP-PrP^∗^-expressing cells were untreated (left) or treated with MβCD (right) for 90 min and then fixed and stained with anti-GFP antibody to detect YFP-PrP^∗^ exposed on the cell surface. (C) Cells were transfected with YFP-PrP^∗^, ER marker CFP-KDEL, and lysosomal marker LAMP1-mCherry and, 48 hr after transfection, were either left untreated (left) or incubated for 6 hr with leupeptin (+LEU) prior to imaging. The boxes indicate the regions that are magnified on the right side of each panel. (D) Images at different time points after transient transfection with YFP-PrP^∗^ in cells stably expressing the Golgi marker, GalT-CFP. (E) An experiment as in (D) imaged for a longer time (see Movie S3). (F) Western blots of N2a cells 48 hr after YFP-PrP^∗^ transfection, 8 hr after 0.5 mM DTT treatment (DTT), or from untransfected and untreated cells (untreated). Approximately equal loading was confirmed by Ponceau S (Pon S) staining. The red asterisks indicate the positions of XBP1 and CHOP. Scale bars, 10 μm. See also Figure S6 and Movie S3.

**Figure S6:**
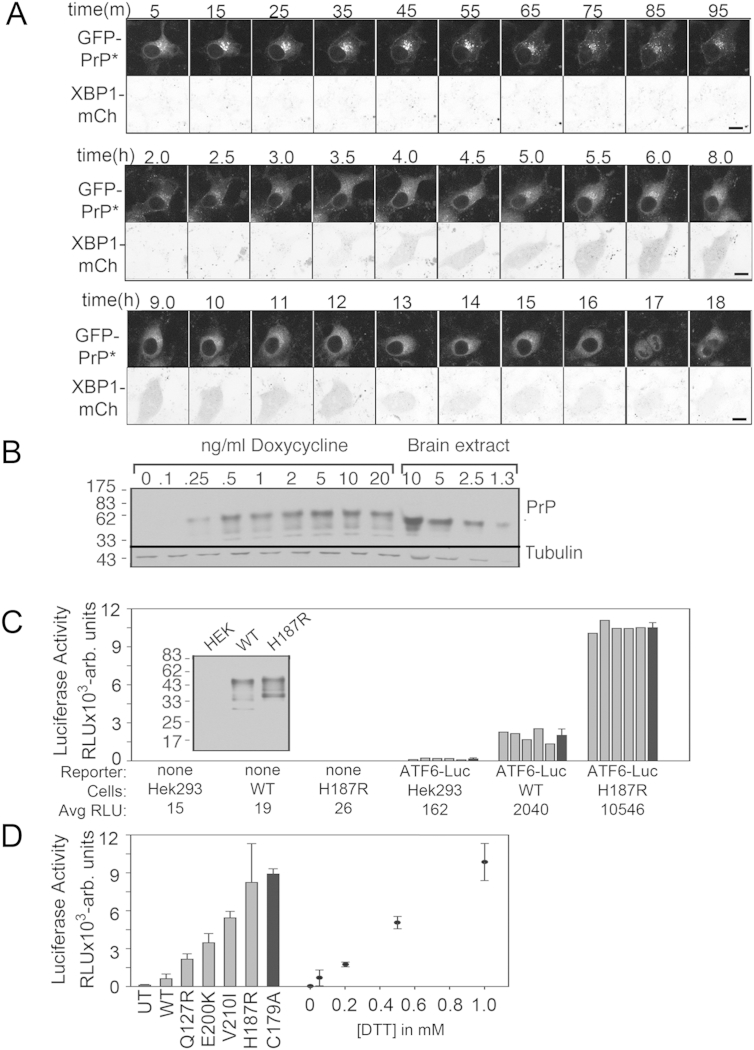
Relationship between RESET and the UPR, Related to Figure 7 (A) Extended data set for Figure 7A. NRK cells stably expressing the XBP1-mCherry UPR reporter and GFP-PrP^∗^ were treated with 0.5 mM dithiothreitol (DTT). Time series were collected over the course of 18 hr starting 5 min after addition of DTT. For each time point, both GFP-PrP^∗^ and XBP1-mCh were imaged. Scale bar, 10 μm. (B) Flp-In T-REx HEK293 cells (Invitrogen) expressing wild-type human PrP at the FRT locus were induced with the indicated concentrations of doxycycline for 24 hr. Total cell lysates were analyzed by immunoblotting with the 3F4 monoclonal antibody, using total hamster brain homogenate as a standard. Loading was controlled by β-tubulin immunoblot. Similar levels of expression of PrP were seen with transient transfection and doxycycline induction in the Flp-In T-REx HEK293 cells. (C) Flp-In T-REx HEK293 cells, either untransfected, or stably transfected with the indicated PrP construct, were transfected with an ATF6-luciferase UPR reporter (or control plasmid), then induced with 5 ng/ml doxycycline for 16 hr. Cells were then assessed for luciferase activity. Five individual wells of cells were measured for each condition (gray bars). The mean of the five replicates (±SD) is shown with the black bars, and indicated below the graph. (D) Flp-In T-REx HEK293 cells, which were either untransfected (UT) or stably transfected with the indicated inducible PrP construct (C179A is YFP-PrP^∗^), were transfected with an ATF6-luciferase reporter. Expression of PrP constructs was induced with 5 ng/ml doxycycline for 16 hr. Triplicate wells were then assessed for luciferase activity (mean ± SD). In parallel, Flp-In T-REx HEK293 cells were transfected with an ATF6-luciferase reporter, treated with the indicated concentrations of dithiothreitol (DTT) for 16 hr, and analyzed in triplicate for luciferase activity (mean ± SD).

Induction of ER stress at a point when most YFP-PrP^∗^ was in the ER triggered RESET of YFP-PrP^∗^ within minutes, whereas XBP1-mCherry was detectably produced starting at 3 hr (Figures 7A and S6A). UPR induction was maximal from between ∼4 and 10 hr, after which it declined. Remarkably, localization of PrP^∗^ began reverting to the ER within ∼3.5 hr, and after 10 hr, PrP^∗^ predominantly localized to the ER. Thus, RESET appears to be most active during the window of time when ER function is compromised and before the cell has compensated via UPR action. After UPR-mediated gene expression begins to restore ER homeostasis, most of the PrP^∗^ is again found to be retained in the ER.

### Degradation of Misfolded GPI-Anchored Proteins in Cells without Exogenous Stressors

Given that misfolded GPI-anchored proteins appear to be poor ERAD clients, we investigated their degradation in the absence of acute ER stress. Three experiments suggested that their degradation culminates in lysosomes by a pathway similar to RESET. First, treatment of YFP-PrP^∗^-expressing cells with MβCD resulted in its trapping at the plasma membrane (Figure 7B). Second, antibody uptake experiments illustrated that, even without ER stress, YFP-PrP^∗^ accesses the extracellular space at least transiently (data not shown). And third, inhibition of lysosomal proteases resulted in YFP-PrP^∗^ accumulation in puncta that colocalized with a lysosomal marker (Figure 7C). Thus, although constitutive degradation of PrP^∗^ occurs at a slower rate in unstressed cells such that most of the YFP-PrP^∗^ is ER localized, the constitutive degradation pathway shares with RESET the trafficking itinerary as well as the final destination.

Insight into the relationship between the stress-induced and steady-state degradation pathways came from careful examination of YFP-PrP^∗^ trafficking upon induction of its expression. We observed that, shortly after transient transfection, most of the newly synthesized YFP-PrP^∗^ was evidently undergoing RESET as judged by its post-ER localization (Figure 7D and Movie S3). At longer times after transient transfection, the cells attained a new steady state in which most of the increased load of PrP^∗^ could be localized to the ER (Figures 7C and 7E and Movie S3). This phenomenon could be explained by the possibility that transient expression of a misfolded protein is itself an ER stressor. Indeed, detecting transcription factors downstream of UPR stress transducers (Figure 7F) or using a luciferase reporter of UPR activation revealed that induction of YFP-PrP^∗^ or any of several other PrP mutants caused ER stress (Figures S6B–6D). The level of luciferase reporter activation was roughly similar to that observed with ∼0.5 to 1 mM DTT (Figure S6D). This explains why, at short times after transfection, YFP-PrP^∗^ undergoes RESET, whereas at later time points, adaptation of cells via UPR induction allows cells to achieve a new steady state in which PrP^∗^ is predominantly localized to the ER.

It is worth noting that, in the adapted state, YFP-PrP^∗^ is still being exported for lysosomal degradation via the secretory pathway. This can be rationalized by considering that binding and release from ER chaperones is in competition with alternative fates (Sekijima et al., 2005), such as binding to export receptors like Tmp21. Thus, the balance may favor chaperones at steady state but may shift strongly toward export when chaperone function is overwhelmed or compromised as during ER stress. Furthermore, even though the cells have adapted to YFP-PrP^∗^ expression via UPR induction, they are nevertheless still susceptible to additional stressors that act globally by perturbing the folding of other proteins, explaining why YFP-PrP^∗^ in adapted cells is rapidly routed for RESET with subsequent DTT or thapsigargin treatment.

## Discussion

Our findings have revealed a previously unappreciated pathway for degradation of misfolded GPI-anchored proteins that represents a protective mechanism to alleviate acute ER stress. By allowing a subset of misfolded proteins to engage cargo receptors, the burden of misfolded proteins in the ER is rapidly reduced and aggregation is avoided. In the case of the GPI-anchored proteins studied here, the primary mechanism of ER retention is an association with calnexin, whereas the primary mechanism of egress depends on the ER export receptor Tmp21. Interestingly, whereas folded GPI-anchored proteins can clearly use Tmp21-independent routes for ER export (Bonnon et al., 2010; Takida et al., 2008), misfolded GPI-anchored proteins seem to be strongly dependent on Tmp21. This may explain the constitutive ER stress phenotype seen in Tmp21-deficient cells of *Drosophila* and yeast (Belden and Barlowe, 2001; Boltz and Carney, 2008; Jonikas et al., 2009) and the early embryonic lethality of Tmp21 knockout mice (Denzel et al., 2000).

The discovery that ER stress triggers the dramatic escape of misfolded, GPI-anchored proteins from the ER to the Golgi was unexpected. Most other reports show that treatment with ER stressors results in tight binding of ER-localized proteins to ER chaperones (Kim and Arvan, 1995; Ma and Hendershot, 2004). The mechanistic basis for this difference is likely to hinge on two major determinants: a misfolded domain and a GPI anchor. Although neither the misfolded domain nor the GPI anchor is sufficient alone, they collude to permit RESET for multiple unrelated proteins through a requisite interaction with Tmp21.

Notably, the folding status of the GPI-anchored protein appeared to influence its dependence on Tmp21 for ER export. Whereas surface localization of wild-type PrP or CD59 was not appreciably affected by Tmp21 knockdown, ER export of the misfolded variants of PrP and CD59 was severely compromised. At present, the basis of this discrimination is not clear and merits further investigation. One possible explanation may be related to the observed ability of Tmp21 to prevent PrP^∗^ aggregation in the ER independently of mediating its export. Tmp21, either directly or in conjunction with other factors, maintains misfolded PrP in a soluble state. Thus, Tmp21 may act as a chaperone to bind misfolded GPI-anchored proteins, preventing inappropriate interactions and ensuring safe passage through the secretory pathway en route to lysosomes.

Tmp21 is a type 1 transmembrane protein that cycles between the ER and Golgi and contains a cytosolic COPII binding signal (Blum et al., 1996; Dominguez et al., 1998; Nickel et al., 1997). Thus, Tmp21-mediated ER export of GPI-anchored proteins could be explained by its ability to bridge the gap between the GPI-anchored clients within the ER lumen and the vesicular trafficking machinery on the cytosolic side. Hence, ER retention of PrP^∗^ in Tmp21 knockdown cells could be due simply to the lack of a suitable export receptor. Although we cannot rule out the alternative explanation that failed export in Tmp21 knockdown cells is a secondary consequence of an altered ER environment, the physical interaction between Tmp21 and PrP^∗^ and previous reports suggesting that p24 proteins facilitate GPI-anchored protein ER export (Bonnon et al., 2010; Schimmöller et al., 1995; Takida et al., 2008) support a more direct mechanism.

It is emerging that GPI-anchored proteins may be refractory to degradation by ERAD (Ashok and Hegde, 2009; this study). This may be because the covalently attached lipid in the lumenal leaflet of the lipid bilayer poses a topologic problem for ERAD machinery. For example, earlier work clearly showed that a PrP mutant (H187R) containing an unprocessed GPI-anchoring signal sequence is a strong degron for ERAD, but the identical protein containing a GPI anchor is degraded in lysosomes (Ashok and Hegde, 2008, 2009). Similarly, the efficient ERAD substrate TCR-α can be converted to a RESET substrate by changing it from a transmembrane protein to a GPI-anchored protein (Suzuki et al., 1991; this study).

The difficulty in degrading misfolded PrP from the ER and its propensity to aggregate there under stress conditions may explain why multiple mechanisms have evolved to prevent its residence in the ER lumen during stress. In addition to RESET, earlier work showed that the efficiency of PrP translocation into the ER is reduced during ER stress (Kang et al., 2006). This attenuation was dependent on the PrP signal peptide and, when bypassed, led to its increased propensity to aggregate inside the ER. Thus, by a combination of both clearance via RESET and attenuation of new entry, aggregation of misfolded PrP is minimized inside the ER lumen. The extent to which these mechanisms apply to other GPI-anchored proteins remains to be investigated in detail.

Stress induction simply by the expression of disease-associated PrP mutants (Figures S6B–S6D), combined with our discovery of a RESET pathway for lysosomal degradation, may explain why most mutant PrPs are found to be only partially retained in the ER (Ashok and Hegde, 2009; Gu et al., 2007; Ivanova et al., 2001). We posit that the constitutive ER stress caused by mutant PrP expression activates RESET, at least partially, leading to mutant PrP egress via the secretory pathway. Indeed, knockdown of Tmp21 leads to increased ER retention of mutant PrP, but not wild-type PrP, at steady state. The net consequence of these effects would be a highly heterogeneous distribution of mutant PrP in the secretory pathway, constitutive ER stress, overall reduced plasma membrane localization, and primarily lysosomal degradation. This is precisely the picture of mutant PrP behavior deduced from multiple earlier analyses (Ashok and Hegde, 2009; Gu et al., 2007; Ivanova et al., 2001; Negro et al., 2001).

The transient appearance of PrP^∗^ on the plasma membrane followed by rapid endocytosis and delivery to lysosomes implicates post-ER quality control pathways for the disposal of misfolded proteins released from the ER by RESET. At present, the mechanisms that permit the cell to distinguish folded from misfolded GPI-anchored proteins at the plasma membrane (or Golgi) are unknown. Although plasma membrane quality control has been described for the integral membrane proteins CFTR (Okiyoneda et al., 2010), an artificially constructed thermolabile substrate (Apaja et al., 2010), and a panel of thermolabile mutant transporters (Zhao et al., 2013), in each case, cargo recognition begins with ubiquitination on the misfolded cytosolic domain. GPI-anchored proteins lack a cytosolically exposed domain. Thus, understanding how noncytosolic defects in proteins can be detected in post-ER compartments remains an important goal for future studies aimed at clarifying the downstream fate of proteins undergoing RESET.

Our studies initially revealed and characterized RESET by using chemical stressors. However, several observations argue for this pathway’s relevance in physiological situations. First, GPI-anchored proteins are abundant and ubiquitous (Ferguson et al., 2009) with many known disease-causing misfolding variants (Grapp et al., 2012; Gu et al., 2007; Ivanova et al., 2001; Nevo et al., 2013). Given that diverse misfolded GPI-anchored proteins undergo RESET in multiple cell types, RESET is likely to be a widely used mechanism for the clearance of misfolded GPI-anchored proteins from the ER. Second, cells commonly experience any of a variety of physiological acute stressors such as viral infection (Diehl et al., 2011), ischema (Perman et al., 2011), and differentiation (Reimold et al., 2001), where RESET may act to ameliorate the imbalance in ER homeostasis prior to UPR-mediated adaptation. This idea is supported by our demonstration that new expression of PrP^∗^ alone, at physiological levels without additional chemical stressors, induces acute ER stress sufficient to induce RESET of PrP^∗^. Subsequently, the UPR is triggered in PrP^∗^-expressing cells, which likely leads to a new homeostasis, as indicated by the eventual retention of most of the PrP^∗^ in the ER. Third, in cells that have adapted to PrP^∗^ expression, the export pathway to lysosomes was utilized at low levels by PrP^∗^ for constitutive degradation.

The rate of engagement of the RESET pathway is predicted to be contingent on the dynamics of interaction between ER resident chaperones and ER export receptors. This is presumably a continuum and is dependent on both the substrate in question and homeostatic state of the ER (Sekijima et al., 2005). In this view, RESET is an enhanced version of a degradation pathway that occurs at more modest levels in the absence of exogenous stressors. This is analogous to other aspects of ER stress responses, where physiologic changes in flux through the ER induce modest changes in the same pathways that were first discovered using artificial reporters and chemical stressors (e.g., ERAD [Bonifacino and Lippincott-Schwartz, 1991] or the UPR [Ma and Hendershot, 2001]). Our conceptual description of RESET, along with the detailed characterization of Tmp21-dependent RESET for GPI-anchored proteins, provides a foundation for future exploration of its different physiologic and pathophysiologic roles.

## Experimental Procedures

Detailed methods are provided in the Extended Experimental Procedures.

### Cells, Plasmids, and siRNA

All experiments were performed in stably transfected normal rat kidney (NRK) cells, unless specified. All cells cotransfected with organelle markers were imaged 48 hr after transient transfection. YFP-PrP was constructed by replacing the endogenous PrP signal sequence (residues 1–22) with the bovine preprolactin-signal sequence to ensure efficient translocation into the ER (Rane et al., 2004) and inserting mYFP into the unique Bsu36I site. Construction of eGFP-CD59, GFP-Thy1, GFP-DAF, and GFP-FolR is described in the Extended Experimental Procedures. Misfolding mutants were derived by site-directed mutagenesis. Tmp21 was depleted by two sequential knockdowns within 48 hr using 50 nM concentration of ON-TARGETplus SMARTpool Rat Tmed10 siRNA and Dharmafect 2 transfection reagent (Dharmacon), and experiments were performed 24 hr after the second knockdown.

### Drug Treatments

Working concentrations are 0.1 μM thapsigargin, 0.5 mM dithiothreitol, 5 mM methyl-β-cyclodextrin, and 1 μM brefeldin A. Lysosomal protease inhibitors refer to 125 μM leupeptin + protease inhibitor cocktail (Sigma P8340) diluted 1:100.

### Microscopy

All fixed and live-cell imaging, excluding FRAP, were performed using the Marianas spinning disk confocal system (Intelligent Imaging Innovations) attached to a Zeiss Observer.Z1 microscope (Carl Zeiss MicroImaging) and analyzed by Slidebook software. FRAP was performed and analyzed with the Zeiss LSM 710 system (Carl Zeiss MicroImaging).

### Biochemistry

For steady-state chase experiments, cells were labeled for ∼10 hr with 100 μCi/ml ^35^S label (NEG072002MC, Perkin Elmer) in Cys-free/Met-free media (D0422, Sigma) supplemented with 10% serum, 2 mM L-Glu, 0.6 μM Cys, and 2 μM Met. Column purifications were performed with the μMACS GFP Isolation Kit (Miltenyi Biotec) using 1% CHAPS, 50 mM HEPES (pH 7.4), and 100 mM NaCl for cell lysis and washes.

Extended Experimental Procedures **Plasmids and Molecular Biology**

The YFP-PrP construct was prepared in a pCDNA3.1-based vector by replacing the endogenous PrP signal sequence (residues 1-22) of wild-type hamster PrP with the bovine preprolactin-signal sequence to ensure efficient translocation into the ER (Rane et al., 2004). The coding sequence for mYFP was then inserted into the unique Bsu36I site, as previously described (Ashok and Hegde, 2009). All of the following YFP-PrP mutants were derived from YFP-PrP by site directed mutagenesis: C179A (to make YFP-PrP^∗^), C179A/S232W (to create a version of YFP-PrP^∗^ where the cleavage of the GPI-anchor signal sequence is blocked, as described [Ashok and Hegde, 2008]), C179A/S232stop (to create a truncated version of YFP-PrP^∗^ lacking the GPI-anchor protein signal sequence), C179A/N181T/N197T (to create a version of YFP-PrP^∗^ in which both N-linked glycosylation sites are removed), D202N and M129V/F198S (to create mutations associated with human prion disease with partial ER retention [Gu et al., 2007; Ivanova et al., 2001]).

GFP-CD59 (C94S) and the human disease related mutant, GFP-CD59 (C89Y), were derived from the wild-type EGFP-CD59 construct, described previously (Nichols et al., 2001), by site directed mutagenesis. YFP-CD3δ was previously described (Lorenz et al., 2006). GFP-Thy1, GFP-FolR, and GFP-DAF were constructed by cloning the Thy1, folate receptor (FolR) and decay accelerating factor (DAF) cDNAs downstream of the rabbit lactase-phlorizin hydrolase signal sequence and mEGFP of the previously described mEGFP-GPI construct (Sengupta et al., 2011). Specifically, each of the cDNAs were inserted into the BsrGI and XhoI restriction sites of the mEGFP-GPI construct using the following cloning primers listed like this: the part that anneals to GFP is in capital letters, the linker is unformatted and contains the restriction sites, and the part that anneals to the cDNA is underlined. For rat Thy1.1 cDNA (gift from Alex Ritter) primers were (forward) CTC GTG TAC AAG gca gga ggc agc cag agg gtg atc agc and (reverse) cct ctc gag tca cag aga aat gaa gtc cg. For human folate receptor 1 cDNA (obtained from DNASU [Cormier et al., 2010; HsCD00044796]) primers were (forward) CTC GTG TAC AAG gca gga ggc agc agg att gca tgg gcc agg a and (reverse) cct ctc gag tca gct gag cag cca cag c. For human decay accelerating factor (DAF) cDNA (obtained from DNASU [Cormier et al., 2010; HsCD00042793]) primers were (forward) CTC GTG TAC AAG gca gga ggc agc gac tgt ggc ctt ccc cca and (reverse) cct ctc gag cta agt cag caa gcc cat gg. Mutations to construct GFP-Thy1 (C28A), GFP-DAF (C81A), and GFP-FolR (C65W) were made by site directed mutagenesis.

The TCRα-GPI cDNA construct was a gift from Carolyn Suzuki (Suzuki et al., 1991). The XBP1-mCherry construct was a gift from Dr. Michael M. Lizardo and Dr. Chand Khanna (Pediatric Oncology Branch, National Cancer Institute, National Institutes of Health).

The following expression constructs used as markers for cellular compartments were described previously: ER-mCFP-KDEL (gift from Dr. Erik Snapp, constructed as previously described for ER-mGFP-KDEL [Snapp et al., 2006]), mCFP-Sec61β (Snapp et al., 2003), Sec13-mCherry [gift from Prabuddha Sengupta, constructed by replacing the GFP from Sec13-GFP (described in Hammond and Glick [2000]) with mCherry], CFP-ERGIC53/p58 (Ward et al., 2001), CFP-GalT (Cole et al., 1996), LAMP1-mCherry and LAMP1-CFP ([LAMP1 with C-terminal fluorescent protein tags] gifts from Dr. George Patterson, constructed exactly as described for PA-GFP-lgp120 [Patterson and Lippincott-Schwartz, 2002]).

**Cell Lines and Culture Conditions**

A clonal line of Normal Rat Kidney (NRK) cells (Hailey et al., 2010) were transfected using Lipofectamine 2000 (Invitrogen 11668), selected with 300 mg/ml Zeocin (Invitrogen R250) for the YFP-PrP constructs or 800 mg/ml G418 (Cellgro 61-234-RG) for YFP-CD3δ and the GFP-CD59 constructs, and sorted by fluorescence-activated cell sorting to enrich for cells that express YFP or GFP. The mouse brain homogenate used to compare relative expression levels of endogenous mouse brain PrP with YFP-PrP^∗^ in stable cells was a gift from Dr. Graham Diering. NRK cells stably expressing LC3-CFP were previously described (Hailey et al., 2010). Cells were maintained in complete culture medium [DMEM (Cellgro 15-013-CV) supplemented with 10% fetal bovine serum (Cellgro 35-015-CV) and 2 mM L-glutamine (Cellgro 25-005-CI)] at 37°C with 5% CO_2_. Unless specified differently, transient transfections of all cell types were done with Lipofectamine 2000, ∼48 hr prior to imaging, steady-state chase or immunoprecipitation experiments.

**siRNA Knockdown of Tmp21**

Tmp21 was depleted by two sequential knockdowns using ON-TARGETplus SMARTpool Rat Tmed10 siRNA (Dharmacon L-092937-01-0005) and Dharmafect 2 transfection reagent (Dharmacon T-2002-02) exactly as recommended by the manufacturer. The second knockdown was performed 48 hr after the first, and experiments were performed 24 hr after the second knock down.

**Antibodies**

Rabbit antisera for GFP and RFP were made as described (Stefanovic and Hegde, 2007) and rabbit antisera for Tmp21 was made by immunizing rabbits with a peptide corresponding to the cytoplasmic tail (CLRRFFKAKKLIE) conjugated to keyhole limpet hemacyanin via the terminal cysteine. The PrP-A antibody was previously described (Rane et al., 2004). The antibody against TCR-α, A2B4, (gift from Carolyn Suzuki) was previously described (Suzuki et al., 1991). CHOP and XBP-1 antibodies were gifts from Linda Hendershot.

Purchased antibodies included anti-calnexin (StressGen SPA-860), anti-Grp94 (StressGen SPA-850), anti-ERGIC53 (Santa Cruz SC-66880), anti-alpha tubulin (Sigma T6199), anti-GAPDH (Santa Cruz SC-20357), Cy5-conjugated anti-rabbit IgG (Jackson ImmunoResearch 111-175-003) and Alexa 488 goat anti-mouse Ab (Invitrogen A11029).

**Drug Treatments**

The following reagents were used at working concentrations as listed, unless specified: 0.1 μM thapsigargin (EMD 586005), 0.5 mM 1,3-dithiothreitol (Roche 10708984001), 125 μM leupeptin (Sigma L5793), 250 nM bafilomycin A1 (LC Laboratories B-1080), 5 mM methyl-β-cyclodextrin (Sigma C4555), 1 μM brefeldin A (Sigma B7651). Lysosomal Protease Inhibitor Cocktail (Sigma P8340) was diluted in media at 1:100.

**Imaging Experiments**

Live cell imaging experiments spanning four hours or less were performed on cells grown in LabTek chambers (Daigger EF8632D) in CO_2_-independent media (Invitrogen 18045088) supplemented with 10% fetal bovine serum at 37°C. For long term imaging experiments over four hours, cells were incubated in complete culture medium (described above) at 37°C with 5% CO_2_ on the microscope stage.

For immunofluorescence of PrP, cells were washed with phosphate-buffered saline (PBS), fixed in PBS containing 3.7% paraformaldehyde for 10 min at room temperature, permeabilized with 0.1% Triton X-100 in PBS, blocked with 10% FBS in PBS (FBP), stained with rabbit polyclonal antibodies at 1:5000 in FBP, washed with FBP and stained with the secondary Cy5-conjugated anti-rabbit IgG (Jackson ImmunoResearch 111-175-003) diluted to 1:500 in FBP, and finally imaged in FBP.

For immunofluorescence of Tmp21 and/or TCR-α, cells were washed with PBS and fixed with a 3:1 methanol-acetone mixture at −20°C for 10 min, blocked with 10% FBS and 5% milk in PBS, stained with A2B4 (for TCR-α) or Tmp21 serum diluted 1:500 in blocking solution for one hour at room temperature. (These steps were performed sequentially when staining both TCR-α and Tmp21). Next, cells were washed 3 times with PBS, and Alexa488 goat anti-mouse (for A2B4) and/or Cy5 goat anti-rabbit (for Tmp21) were each used at 1:1000 in the blocking solution for 1 hr at room temperature. Cells were washed 3 times with PBS and imaged in PBS.

For antibody uptake analyses, cells were first incubated with primary antibody at 1:5000 in DMEM with the indicated drugs for 90 min, then washed, fixed, permeabilized, stained with secondary Ab, and imaged exactly as above. To specifically probe cell-surface exposed YFP-PrP^∗^, cells were not permeabilized.

Fixed and live cell images were acquired with a Marianas spinning disk confocal system (Intelligent Imaging Innovations) attached to a Zeiss Observer.Z1 microscope with a 63 × 1.4 NA Plan-Apochromat oil objective (Carl Zeiss MicroImaging, Inc.) using 445 nm, 488 nm, 515 nm and 561 nm diode lasers to image ECFP, EGFP, EYFP and mCherry respectively. Images were acquired and analyzed using Slidebook 4.2 (Intelligent Imaging Innovations).

Fluorescence recovery after photobleaching (FRAP) was performed and analyzed with the Zeiss LSM 710 system (Carl Zeiss MicroImaging, Inc.).

**Biochemical Analyses**

All experiments were performed in stably transfected NRK cells cultured in 6-well dishes to ∼60%–80% confluency. For steady-state chase and co-immunoprecipitation (co-IP) experiments, cells were labeled for 10-12 hr with 100 μCi/ml ^35^S label (Perkin Elmer NEG072002MC) in Cys-free/Met-free media (Sigma D0422) supplemented with 10% fetal bovine serum (FBS), 2 mM L-Glu, 0.6 μM Cys and 2 μM Met. For the chase, cells were washed with PBS to remove the label and incubated with complete culture medium (described above). Drugs were added at the time of chase. Finally, cells were washed in PBS and solubilized in 150 ul of 1% SDS, 0.1 M Tris, pH 8, by boiling/vortexing. Lysates were diluted 10-fold in IP buffer (1% Triton X-100, 50 mM HEPES, pH 7.4, 100 mM NaCl). For non-denaturing co-IP, cells were harvested in co-IP buffer (1% CHAPS, 50 mM HEPES, pH 7.4, 100 mM NaCl) on ice and clarified by centrifugation in a microcentrifuge. Immunoprecipitation was done by incubating lysates with specified rabbit polyclonal antibodies and protein A gel (BioRad 153-6154). For sequential IPs, non-denaturing IPs were performed, immunoprecipitated protein complexes were denatured in 1% SDS, 0.1 M Tris, pH 8, and then diluted 10-fold in IP buffer before the second round of IP. Proteins were separated on Tris/Tricine gels. The gels were dried and exposed on Biomax MR film (Kodak Cat 895-2855). To verify equal starting material and equal recovery of the antibody complexes, diluted lysates were also analyzed, and gels of the IP were stained with Coomassie blue to visualize antibody light and heavy chains.

GFP pull-downs were performed with the μMACS™ GFP Isolation Kit (Miltenyi Biotec 130-091-288) exactly according to manufacturer’s recommendations except that 1% CHAPS, 50 mM HEPES, pH 7.4, 100 mM NaCl was used for cell lysis and washes.

**ATF6-Luciferase Assay**

The ATF6-luciferase construct was obtained from Ron Prywes (Shen and Prywes, 2005) and encodes 5 tandem ATF6 promoter elements upstream of the luciferase open reading frame. Flp-In T-REx HEK293 cells were from Invitrogen. For inducible expression in these cells, the desired open reading frame (either wild-type human PrP or its various mutants) was subcloned into the pcDNA™5/FRT/TO vector (Invitrogen V6520-20) at the HindIII and BamHI sites. Transient transfection with these constructs resulted in little or no expression unless induced with doxycycline. It was important to use media containing serum that had either been stripped with activated charcoal, or certified to be doxycycline-free. For generation of stable cells, the pcDNA™5/FRT/TO plasmids were co-transfected with pOG44 (encoding the Flp recombinase; Invitrogen V6005-20) into the Flp-In T-REx HEK293 cells. Stable integrants were selected for as recommended by the manufacturer.

Luciferase assays employed transient transfection with the ATF6-luciferase reporter and analysis of luciferase activity using luciferase assay reagents (Roche 11 669 893 001) as recommended by the manufacturer. Assays were typically performed in 96 well plates directly on transfected cells. Briefly, the culture medium was aspirated, and the luciferase reagents (which contain a cell lysis reagent) were added directly to the well. Readings were performed using a luminescence plate reader.

## Author Contributions

P.S.-K., R.S.H., and J.L.-S. conceived and directed the project and wrote the manuscript. P.S.-K. designed and carried out most of the experiments. M.A. assisted with molecular biology and microscopy experiments. S.B. performed the ATF6-luciferase assays to analyze the UPR response to PrP mutants, and E.I. provided characterization of the disease mutant PrP cell lines.

## Supplemental Information

Movie S1. YFP-PrP^∗^ Rapidly Relocalizes in Cells Treated with Thapsigargin, Related to Figure 1 Shown is a field of NRK cells stably expressing YFP-PrP^∗^ (YFP-PrP^∗^-cells). Time-lapse images were collected in 2 min intervals spanning 1 hr starting immediately after treatment with 0.1 μM thapsigargin. Time point annotations are shown in the top left corner, and a 10 μm scale bar is in the bottom left corner of the movie.

Movie S2. YFP-PrP^∗^ Rapidly Relocalizes in Cells Treated with Dithiothreitol, Related to Figure 1 Shown is a field of NRK cells stably expressing YFP-PrP^∗^ (YFP-PrP^∗^-cells). Time-lapse images were collected in 2 min intervals spanning 1.5 hr starting immediately after treatment with 0.5 mM dithiothreitol. Time point annotations are shown in the top left corner, and a 10 μm scale bar is in the bottom left corner of the movie.

Movie S3. Acutely Expressed YFP-PrP^∗^ Undergoes RESET, Related to Figure 7 Shown is an NRK cell imaged for up to 15 hr after transient transfection with GFP-PrP^∗^. Time-lapse images were collected at 30 min or 1 hr intervals, as indicated. Time point annotations and a 10 μm scale bar are displayed.

Document S1. Article plus Supplemental Information
