# Supplementary figures and images for: ER Stress-Induced Clearance of Misfolded GPI-Anchored Proteins via the Secretory Pathway
Source: Cell. 2014 Jul 31;158(3):522–33. doi: 10.1016/j.cell.2014.06.026 (PMC4121523; doi:10.1016/j.cell.2014.06.026)

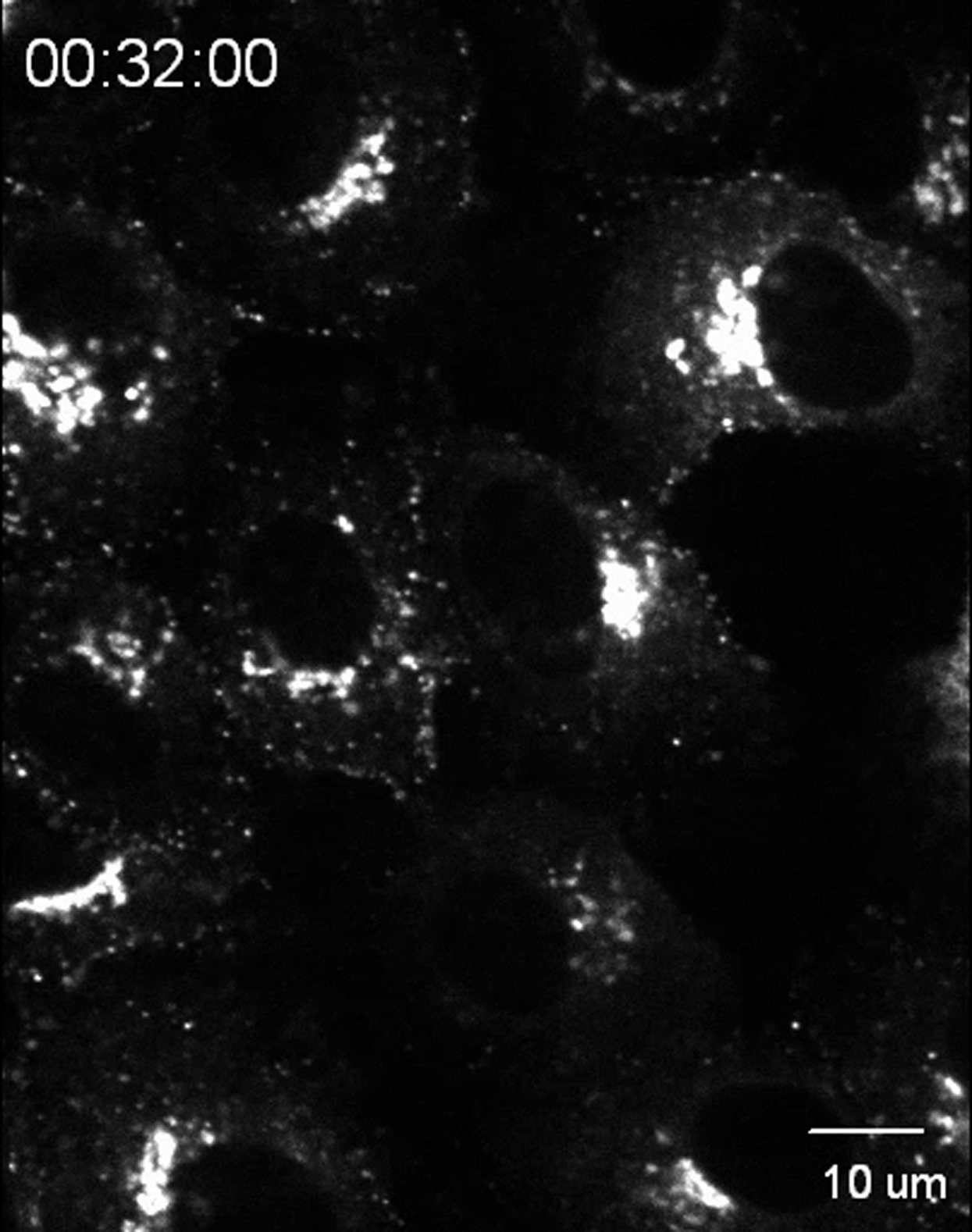

Supplement: Movie S1. YFP-PrP∗ Rapidly Relocalizes in Cells Treated with Thapsigargin, Related to Figure 1 — Shown is a field of NRK cells stably expressing YFP-PrP∗ (YFP-PrP∗-cells). Time-lapse images were collected in 2 min intervals spanning 1 hr starting immediately after treatment with 0.1 μM thapsigargin. Time point annotations are shown in the top left corner, and a 10 μm scale bar is in the bottom left corner of the movie. [file mmc1.jpg]

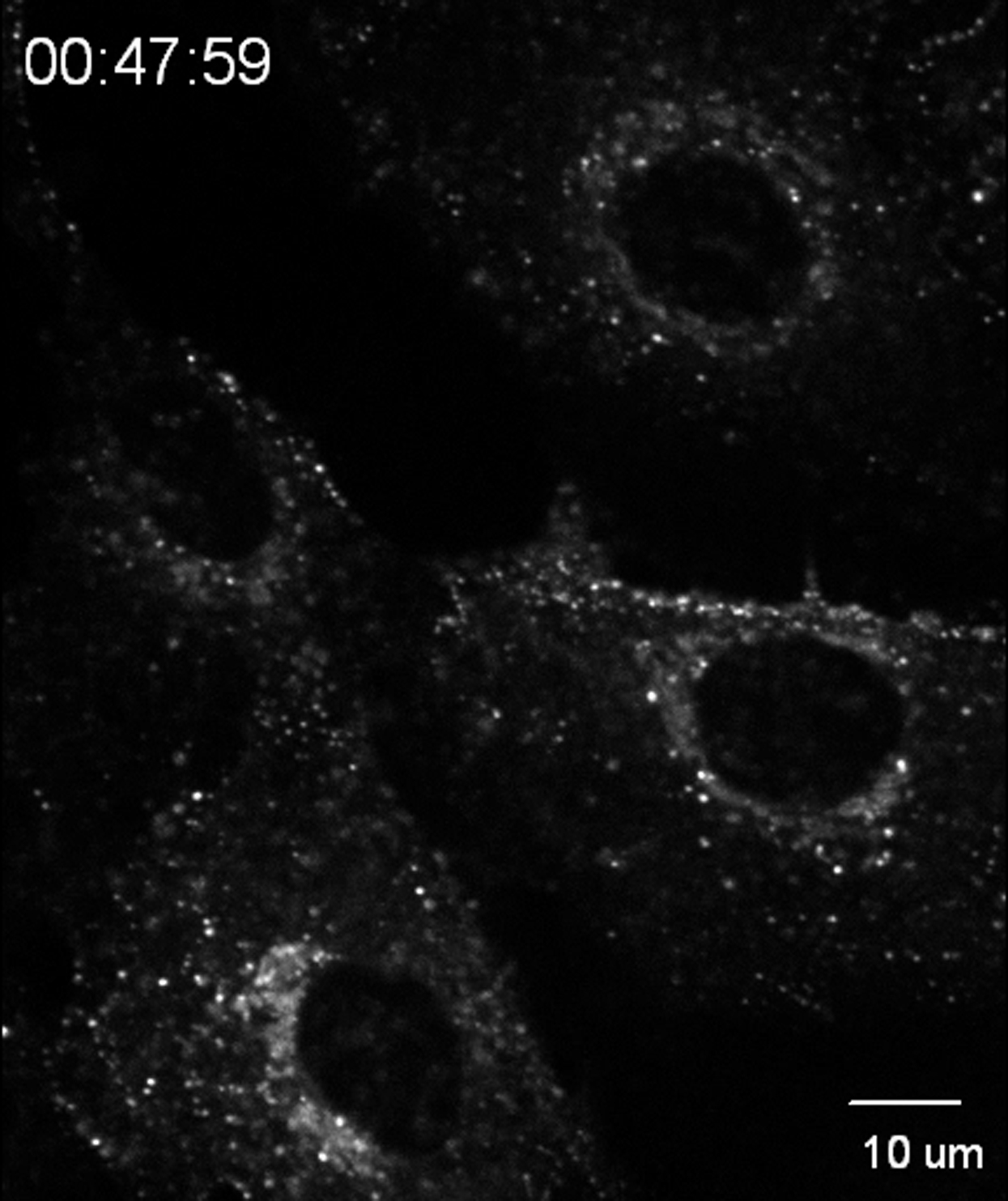

Supplement: Movie S2. YFP-PrP∗ Rapidly Relocalizes in Cells Treated with Dithiothreitol, Related to Figure 1 — Shown is a field of NRK cells stably expressing YFP-PrP∗ (YFP-PrP∗-cells). Time-lapse images were collected in 2 min intervals spanning 1.5 hr starting immediately after treatment with 0.5 mM dithiothreitol. Time point annotations are shown in the top left corner, and a 10 μm scale bar is in the bottom left corner of the movie. [file mmc2.jpg]

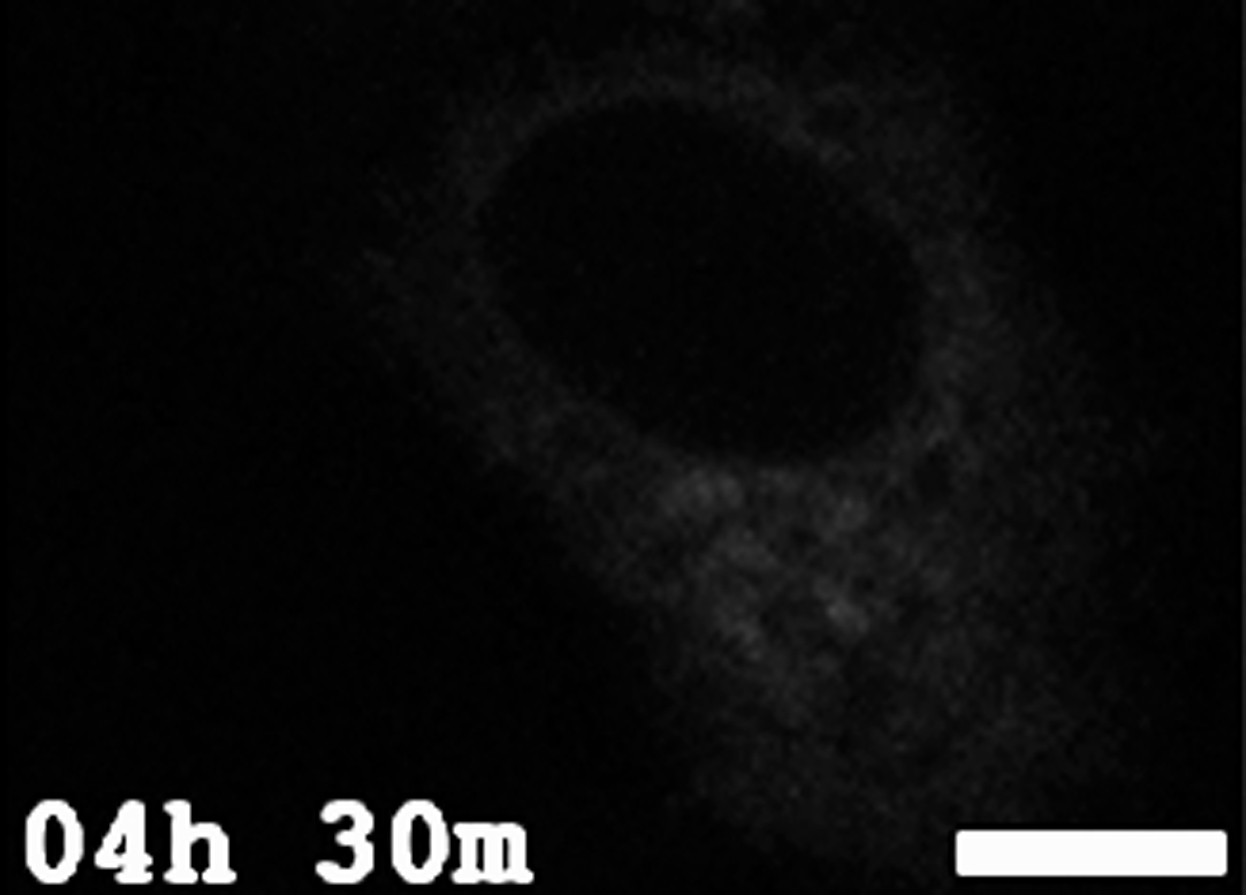

Supplement: Movie S3. Acutely Expressed YFP-PrP∗ Undergoes RESET, Related to Figure 7 — Shown is an NRK cell imaged for up to 15 hr after transient transfection with GFP-PrP∗. Time-lapse images were collected at 30 min or 1 hr intervals, as indicated. Time point annotations and a 10 μm scale bar are displayed. [file mmc3.jpg]
